# A Review on Halide Perovskite-Based Photocatalysts: Key Factors and Challenges

**DOI:** 10.1021/acsaem.2c02680

**Published:** 2022-12-08

**Authors:** Filipp Temerov, Yasmine Baghdadi, Ed Rattner, Salvador Eslava

**Affiliations:** † Department of Chemical Engineering, Imperial College London, LondonSW7 2AZ, United Kingdom; ‡ Department of Chemistry, University of Eastern Finland, JoensuuFI-80101, Finland

**Keywords:** photocatalysis, photocatalytic CO_2_ reduction, halide perovskites, sustainable energy, solar fuels, design of photoreactors

## Abstract

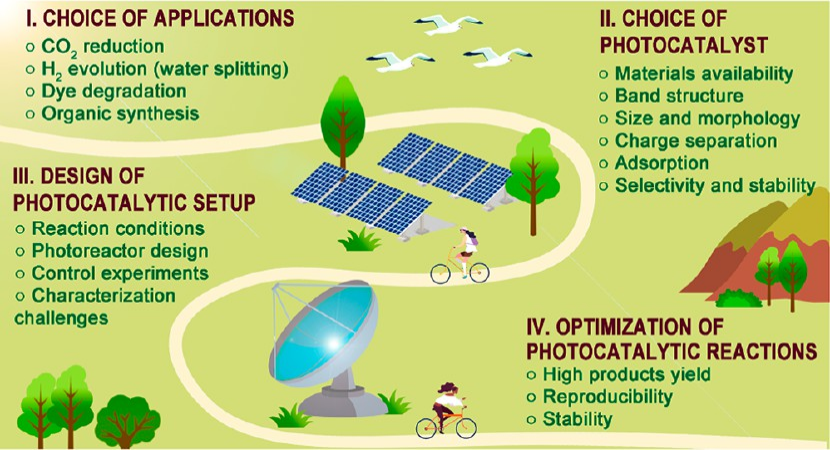

A growing number of research articles have been published on the use of halide perovskite materials for photocatalytic reactions. These articles extend these materials’ great success from solar cells to photocatalytic technologies such as hydrogen production, CO_2_ reduction, dye degradation, and organic synthesis. In the present review article, we first describe the background theory of photocatalysis, followed by a description on the properties of halide perovskites and their development for photocatalysis. We highlight key intrinsic factors influencing their photocatalytic performance, such as stability, electronic band structure, and sorption properties. We also discuss and shed light on key considerations and challenges for their development in photocatalysis, such as those related to reaction conditions, reactor design, presence of degradable organic species, and characterization, especially for CO_2_ photocatalytic reduction. This review on halide perovskite photocatalysts will provide a better understanding for their rational design and development and contribute to their scientific and technological adoption in the wide field of photocatalytic solar devices.

## 1 Introduction

Today, clean energy production is often touted as one of the biggest challenges of the next 20 years. Clean energy is urgently needed to mitigate climate change, that has been exacerbated by the use of fossil fuels to power our industries and urban areas during decades. Indeed, the Paris Agreement on Climate Change and the 26th UN Climate Change Conference of the Parties highlighted the need to move from fossil fuels to renewable sources.^1,2^

Solar energy is a promising and abundant clean energy source, and the technologies to harvest it and store it are rapidly developing. For example, solar energy can be harvested by solar panels and stored in batteries. However, conventional lithium batteries have energy densities of 1 MJ kg^–1^, in comparison with 55 MJ kg^–1^ in fuels such as methane.^3^ Alternatively, solar energy can also be stored in fuels such as hydrogen and hydrocarbons instead of in batteries. To achieve so-called solar fuels, it is necessary to drive reactions which can take up energy (endergonic reactions), so that solar energy can be stored in the bonds created between molecules. An approach to directly produce solar fuels is photocatalysis, that stores the solar energy in fuels involving semiconductors in direct contact with the reactants of the endergonic reactions. The application of photocatalysis to the field of solar energy is inspired by natural photosynthesis, where plants, under sunlight, reduce CO_2_ and produce glucose. Photocatalytic reduction of CO_2_ is practically the reverse of CO_2_ combustion, and it has the potential to ensure high solar energy storage capabilities, making the most of properties inherent to carbon fuels. Photocatalysis of water (H_2_O) and CO_2_ can result in the following solar fuels and feedstocks: H_2_, CO, methanol (CH_3_OH), formic acid (HCOOH), methane (CH_4_), or C_2+_ products, or useful combinations thereof, such as syngas (CO + H_2_).

Concurrently, photocatalysis is also used as means of water purification in environmental applications. With a photocatalyst, light can break down organic dyes and pollutants into harmless CO_2_ or mineralized carbon such as carbonates. This method holds a comparative advantage against traditional ways of purifying water as it does not generate large volumes of waste material like flocculation, sedimentation, and filtration do. For further reading on photocatalytic water purification, we recommend this review by Chong et al.^4^ In addition, photocatalysis tends to have low operating costs since the reaction can take place under mild operating conditions of pressure, temperature, and volume, and it leverages the solar energy, with great potential in developing countries.

Regardless of the chosen application, photocatalysts need to have specific characteristics that are further detailed in this article, but as a whole, they need to be good absorbers of the solar spectrum for practical applications and semiconductors to generate separated charges with the potential to drive redox reactions. Metal oxides such as titanium dioxide (TiO_2_), zinc oxide (ZnO), α-Fe_2_O_3_, and WO_3_ have been studied for photocatalysis since the inception of the field by the seminal paper of Fujishima and Honda.^5^ The drawback has been that, despite their cost effectiveness and stability, they suffer from poor solar absorption and high recombination of photoinduced charges. Some of the most successful photocatalysts, such as TiO_2_ and ZnO, and even SrTiO_3_:Al, recently used in a 100 m^2^ scale plant,^6^ can only absorb in the ultraviolet (UV) range. As such, visible light photocatalysts have been much sought after as a means of maximizing use of the solar spectrum.

Following from binary metal oxides, oxide perovskites have also been investigated for their potential use as a photocatalyst. They follow a general cubic structure of ABO_3_, where O^2–^ is an oxide anion, and A^2+^ and B^4+^ are divalent and tetravalent metallic cations. From there, it was a small leap until halide perovskites (HPs) took the spotlight, being materials with the same structure, but O^2–^ is replaced with a halide anion, X^–^ (X= Cl^–^, Br^–^, I^–^), A is a monovalent cation A^+^, and B is a metal divalent cation B^2+^, often Pb^2+^ or Sn^2+^. HPs have been one of the fastest-growing areas of research after they showed high performances in solar cells, currently reaching a power conversion efficiency above 25%.^7^ They have been shown to have excellent light absorption capabilities, long charge-carrier diffusion lengths, and high extinction coefficients, which means they can effectively absorb light and transport the photoinduced charge carriers relatively long distances (μm) to reach surface sites for redox reactions.^8^ They have been shown to be easily tunable, which allows band structure tailoring to fit specific applications. They are relatively cheap, and, when considering the new lead-free halide perovskites, environmentally friendly. However, they are not without their drawbacks, which include low thermal stability, especially when A^+^ is an organic cation (often CH_3_NH_3_^+^ or CH(NH_2_)^+^), low moisture stability, and high toxicity when prepared with Pb^2+^. For further detailing of this, we refer the reader to Section 4.1 on stability.^9^ Often, in photocatalytic applications, they must be in contact with water, and research is being done both through the lenses of improving stability or optimizing conditions so that the degradation is minimal. Other characteristics that bring HPs to the forefront of photocatalytic research are their tunable band gap by halide composition or doping.

This work will explain why HPs have risen through the ranks to become one of the photocatalytic materials with the most potential. By examining different synthesis methods and how they affect the structure and properties of HPs, we will draw conclusions on the advantages and disadvantages for specific applications. Further, we will define key factors influencing performance that explain the reasons for the trends observed in the literature, namely stability, band structure, size, morphology, charge separation capability, sorption behavior, and selectivity. Finally, we will delve into the challenges in working and characterizing HPs and important considerations to have when working in photocatalysis, shedding light on promising strategies to achieve an agreed-upon standard of performance.

## 2 Background: Mechanism and Applications

Photocatalysis is dependent upon the use of a semiconductor. On a given semiconductor material, electrons populate the valence band (VB) below the Fermi level. Energy levels which exist above the Fermi level, generally defined as the conduction band (CB), remain empty at ground state. Upon light excitation, electrons may transition from the VB to the CB. Conductors, such as most metals, have no gap or very small gap between these two bands because VB and CB overlap. Conversely, insulators have a large energy gap between the VB and CB (termed band gap). A semiconductor generally sits somewhere in the middle, in which photons with energy corresponding to UV or visible light are able to supply energy to excite electrons into the CB, leaving electron vacancies in the VB, commonly referred to as holes.

**Figure 1:**
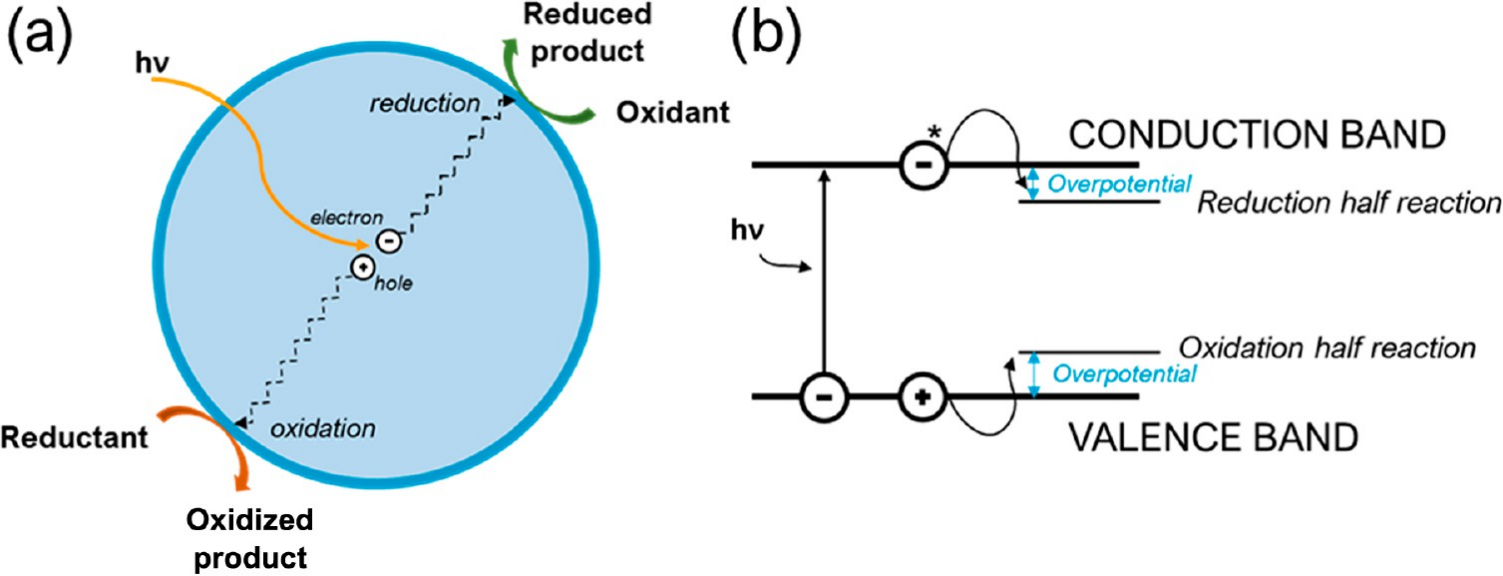
(a) Schematic showing how the absorption of a photon of energy *hv* leads to the separate charge carriers being used for photocatalytic reactions. (b) Schematic showing the thermodynamic (the straddling of the reaction potentials) and kinetic requirements (the overpotential needed as a driving force) for photocatalysis upon the absorption of a photon by a heterogeneous photocatalyst.

Broadly summarized, the mechanism of photocatalysis can be described with pseudochemical reactions that illustrate the mechanistic sequence of the process. The principle that underpins photocatalysis is that, through the photovoltaic effect, a photon with energy larger than the band gap can be absorbed by a semiconductor (SC) and promote an electron from the VB to the CB (eq 1). This means that light can be used to generate charge carriers and provide energy for reactions. Figure 1a illustrates what happens under illumination. A photon is absorbed by a semiconductor (SC), creating an exciton, an electron–hole pair. These can separate and diffuse to the surface. At surface sites, an excited electron can be used to reduce an oxidant and the positively charged hole can accept an electron from a reductant and oxidize it (eqs 2 and 3). However, charges may end up recombining and not being used productively, producing either excess heat or re-emitting light, as showcased in eqs 4 and 5:
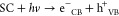
1
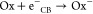
2
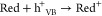
3
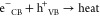
4
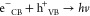
5

There are also constraints to this process: for both oxidation and reduction to be thermodynamically feasible on a single photocatalyst, the band gap must straddle the oxidation and the reduction potentials. This means that only photons whose energy is larger than the band gap can be used to catalyze the target reactions. This implies a trade-off, a large band gap allows for flexibility and for a wider range of catalyzed reactions, as well as a large overpotential driving force, which is needed to drive the reaction rate, but it also means that a smaller part of the whole spectrum of light is used. Optimization and tuning of the band gap is commonly undertaken to achieve higher efficiency or production, and HPs are a material in which this can be done relatively easily. (Figure 1b).

Another important step in photocatalysis, as with all heterogeneous catalysis, is mass transport. Photocatalytic reactions are catalyzed on the surface and for that to happen reactants need to adsorb and products need to desorb. Product desorption is important both to prevent catalyzed back reactions and to free up catalytic adsorption sites for new reactants. An optimal heterogeneous catalytic system operates under reaction control (assuming safety conditions are met), so it is important to design reactor systems that allow the reaction to occur under mass transfer control. This is further discussed under reactor and system design (Section 5.2).

To better understand energetic constraints to the process, one can pinpoint six avenues for energy loss, identified by Stolarczyk et al. as follows:^10^

(1) Below band gap photons. A loss that stems from the use of semiconductors. The sun emits light as a spectrum, from low energy IR to high energy UV. Photons with energy below the band gap cannot be absorbed, meaning that there is a cutoff below which all solar energy is intrinsically unused. Plasmon-induced hot electron transfer is one way to try to mitigate this loss. It can occur when a metal nanoparticle that has been deposited on a photocatalyst absorbs photons with lower energy than the band gap of the semiconductor is attached to. This can lead to an electron being injected into the CB of said semiconductor.^11^ Another of these strategies is upconversion–with the aid of mid band gap energy levels, electrons can be excited in a two-step process absorbing two photons with energy lower than the band gap, in a stepwise process. However, while upconversion increases energy utilization, the existence of energy states in the band gap can also be counterproductive as they can trap charges and act as recombination centers.^12^
(2) Heat loss. Heat loss is the process of energy released as heat. It happens when a semiconductor photocatalyst absorbs an above-band gap photon, and then the corresponding electron is promoted to a level higher than the bottom of the CB: this is called a hot charge carrier. The electron can then collapse to the more stable lowest CB level and release the excess energy as heat.^13^
(3) Charge recombination. Charge recombination is arguably the single most difficult problem in photocatalysis. Charge carriers are extremely short-lived. They often quickly recombine and release the energy either as heat (nonradiative recombination) or re-emit a photon (radiative recombination). Both types represent major energy losses in photocatalysis, and a lot of work is focused on trying to separate the charges as to avoid recombination.
(4) Overpotential. Overpotentials, here defined as the potential differences between the CB edge and the reduction potential (CB-E_red_) and between the VB edge and oxidation potential (VB-*E*_ox_), are another form of energy loss. A large overpotential can increase the charge transfer rate, decreasing the effect of charge recombination, but it does so at expense of extra energy loss. This is exacerbated by the need to use a larger band gap semiconductor to increase the overpotential, for the same pair of photocatalytic half-reactions.
(5) Back reactions. In some cases, the products of photocatalysis are also susceptible to back reactions. Reverse reactions are also thermodynamically feasible in most cases, and some back-formation is to be assumed. This is more prevalent in the case of solar fuel production than for organic pollutant degradation.
(6) Product separation and postprocessing. This is another aspect more closely related to solar fuel production. In photocatalytic setups where specific products are needed, there is the postprocessing energetic cost of separating them. In photocatalysis, both the reduction and the oxidation are catalyzed in the same reactor compartment, resulting in a mixed outlet stream, complete with unreacted precursors. This is a lesser concern for oxidative degradation, where in most cases the final products, due to their harmless nature, do not need to be separated from the reaction medium.

The many energy pitfalls in photocatalysis could potentially discourage uptake of it, but the big advantage is that solar energy is renewable, and that any amount that offsets its own energetic cost of production will be a net gain in the overall energy landscape. To understand how photocatalysis can be used in a productive way, and more specifically, how HPs can be used as effective photocatalysts, we must delve into the applications of the field.

It is also important to understand how to assess photocatalytic performance, that is their metrics. If the desired application is pollutant degradation, this is often reported as percentage concentration decrease, as a fraction of the initial concentration of the target compound. For all other photocatalytic applications, which are the main focus of this review, this is often reported as molar production, and then normalized by relevant conditions, such as mass, time or irradiation area. The result is that performance is often quoted in μmol g^–1^ h^–1^. It deals with amount of product (often low, so in the μmol range), and it is normalized by the time of the reaction and the mass of catalyst used. Variants of this include the time-normalized production (μmol h^–1^) and mass-normalized production (μmol g^–1^), but by far the most standard and uniformly used is the double-normalized value using μmol g^–1^ h^–1^. For surface-mounted catalysts, when evenly distributed, μmol m^–2^ h ^–1^ has sometimes been used.

Another important metric is photoluminescence quantum yield (PLQY). A lot of research on HPs has been derived from light-emitting diode (LED) applications, where the goal is for carriers to recombine radiatively to emit light. In these cases, PLQY should be as high as possible. However, in photocatalysis, there are competing effects that do not allow PLQY to be used as an effective metric. On one hand, having crystals of lower quality decreases PLQY and impairs photocatalytic performance; on the other hand, charge separation, in a heterojunction decreases PLQY but improves photocatalytic performance. It is important to understand these in context, and due to the evolution of the field from light emitting applications, especially on the synthesis side, PLQY is still very often quoted as a metric. We are choosing to report it at times, especially when it can be used as a matter of comparison for improved charged separation.

### 2.1 Hydrogen Production

The first route for photocatalytic hydrogen (H_2_) production was H_2_O splitting, which is the decomposition of H_2_O into H_2_ and oxygen (O_2_). However, HPs tend to be extremely unstable in the presence of moisture, so most advances in hydrogen production have been part of the process called acid splitting, consisting of the reactions in eqs 6–9. In 2017, Park et al. showed that methylammonium lead iodide (MAPbI_3_) can be kept in acid–base equilibrium in a hydroiodic acid (HI) aqueous solution, and can photoreduce HI into H_2_ and I_3_^–^,^14^ extending the large interest in HPs from photovoltaics to heterogeneous photocatalysis:
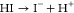
6
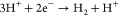
7
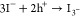
8
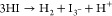
9

**Figure 2:**
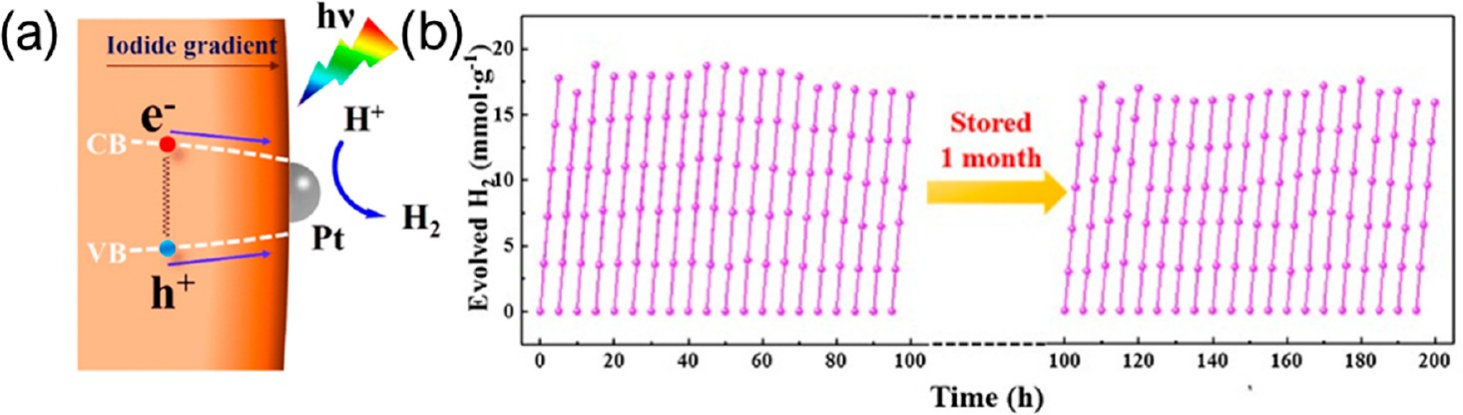
(a) Halide gradient was proposed to drive the charges to the surface, leading to high levels of H_2_ production. Reproduced from ref (16). Copyright 2018 American Chemical Society. (b) Black phosphorene-MAPbI_3_ catalyst shows high production over 100 h of illumination, with performance maintained after 1 month of storage. Reproduced with permission from ref (17). Copyright 2019, Elsevier.

Wu et al. further developed this approach by mounting MAPbI_3_ on reduced graphene oxide (rGO), increasing evolution rate by 67 times and showing no decrease in activity after 200 h of operation.^15^ This highlights the advantage of complex catalyst architectures, further discussed in Section 4. Other improvements include the use of a mixed-halide perovskite with platinum cocatalyst nanoparticles (NPs) MAPbBr_3–*x*_I_*x*_/Pt. They describe a band gap funneling which drives the charges to the surface due to a halide gradient. The presence of Pt centers then helps decrease charge recombination, leading to 2605 μmol g^–1^ h^–1^ of H_2_ and no significant reduction of activity over 6 cycles and 30 h of operation.^16^ The effect of the halide gradient is illustrated in Figure 2 a, causing a narrowing of the band gap. Black phosphorene has also been used with MAPbI_3_ as an electron transport layer to achieve 3742 μmol g^–1^ h^–1^ and excellent stability, as seen in Figure 2 b, where activity is maintained even after 20 cycles and 1 month of storage.^17^

**Figure 3:**
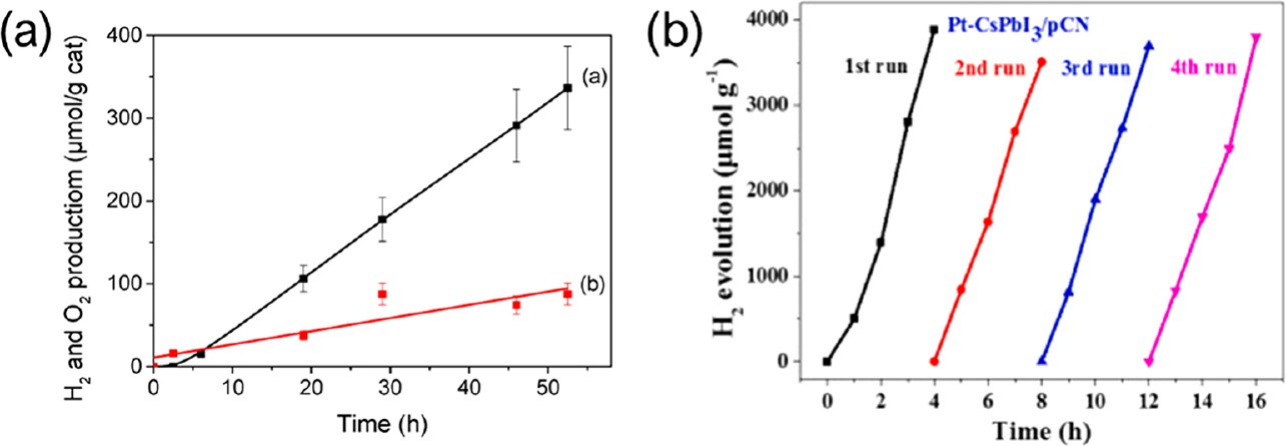
(a) Production of H_2_ (black) and O_2_ (red), showing less than stoichiometric O_2_ from H_2_O splitting. The formation of peroxides was ruled out, so the authors could not explain the disparity. No products were observed in the absence of water. Reproduced with permission from ref (18). Under a CC license, 2020, MDPI. (b) Production of H_2_ over four cycles for a lead halide perovskite, showing remarkable stability. Reproduced with permission from ref (19). Copyright 2021, Elsevier.

Recently, some work has been done on photocatalytic H_2_O splitting using HPs or composites thereof. The H_2_O splitting reactions are stated in eqs 10–12, with the oxidation and reduction reactions with the photoinduced charges (holes and electrons) and the full reaction represented. This is a much newer subset of this application, as HPs tend to be very unstable in the presence of water, but some researches are trying to find routes to make this possible. Garcia et al. used a MA_2_CuCl_2_Br_2_ catalyst to split H_2_O in the gas-phase with 100% humidity, achieving 24 h of operation (Figure 3a).^18^ They also showed that it is also achievable with traditional lead HPs, but with far lower production, at 0.11 μmol g^–1^ h^–1^. It appears that the superior stability of MA_2_CuCl_2_Br_2_ was the main driver for it. CsPbI_3_ and protonated graphitic carbon nitride were combined to split H_2_O in the liquid phase, reaching 242.5 μmol H_2_ g^–1^ h^–1^, which then increased over 3-fold when Pt was used as a cocatalyst.^19^ Interestingly, it achieved no loss of production over 4 cycles and 16 h, shown in Figure 3b. Further, some (low) levels of H_2_ production from water splitting are sometimes observed in experimental setups designed to reduce CO_2_:
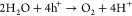
10
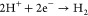
11
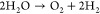
12

### 2.2 CO_2_ Reduction

Carbon valorization has been one of the main focuses of photocatalytic research, trying to convert CO_2_ to higher value chemicals, either CH_4_ for fuel use, or to chemical feedstocks such as CO or CH_3_OH. The CO_2_ reduction with photoinduced electrons needs to be balanced with an oxidation reaction with the photoinduced holes. In order to do this as cheaply as possible, the reductant is often water, which can also act as proton source for hydrocarbon formation. Some reports have used hole sacrificial agents, also called hole scavengers, to focus their research on the CO_2_ reduction. Hole sacrificial agents, such as triethanolamine, are substances that easily oxidize with the photoinduced holes, improving the lifetime of the photoinduced electrons for the reduction to take place. However, for the development of carbon valorization, it is key that the cost of the reductant does not compromise the economic profit of the process. Some of the most important reactions for CO_2_ reduction, as well as the water oxidation reaction which balances the process, are stated in eqs 13–22.^20^ All potentials are stated against the standard hydrogen electrode (SHE) at pH 7:
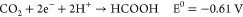
13
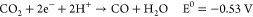
14
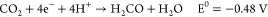
15
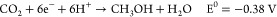
16
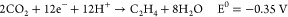
17
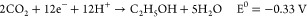
18
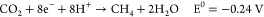
19
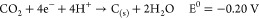
20
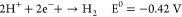
21
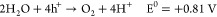
22

When considering what CO_2_ reactions are possible, it is necessary to consider the limitations inherent to this process. First, for reaction on a single semiconductor photocatalyst, the band gap must straddle the oxidation and reduction potentials so both photoinduced electrons and holes are used. Larger overpotentials will drive kinetic rates and could change selectivity. Second, charge carriers are short-lived. This means that reactions which use large numbers of excited electrons are very difficult to achieve (for this reason, it is very rare to see direct reduction to C_2_ products). This pushes selectivity toward reactions with a lower number of electrons taking part. In several reactions, these two effects counteract each other and determining the balance is essential to explain behaviors observed in experiments. It has been shown that when charge transport layers are added to more effectively separate the charges, this shifts selectivity toward different products (higher number of electrons and higher potential).^21^ Another aspect to consider is the desorption of products, different products may have different desorption energies, leading to a slower freeing of catalytic sites, which can slow down the reaction rates overall.^22^

**Figure 4:**
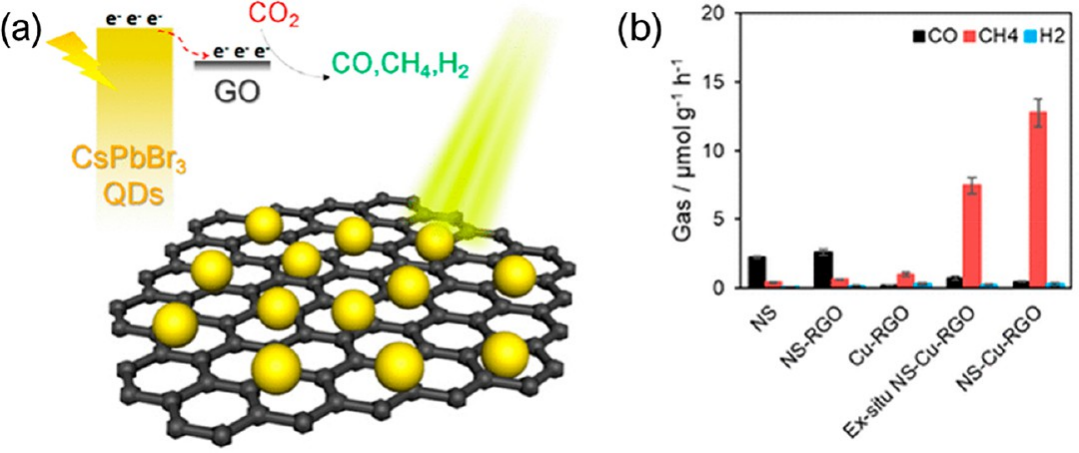
(a) Illustration of CsPbBr_3_ QDs deposited on high surface area graphene oxide, with a diagram showing the energy step the electrons take as they migrate to GO. Reproduced from ref (24). Copyright 2017, American Chemical Society. (b) Photocatalytic production of CsPbBr_3_ nanosheets, and production with the addition of transport layers and metal cocatalyst. The selectivity shifts from CO to CH_4_. Reproduced from ref (21). Copyright 2020, American Chemical Society.

HPs have advantages such as a tunable band gap to straddle the CO_2_ reduction and H_2_O oxidation on the same photocatalyst. Still, currently, most state-of-the-art research focuses on complex catalyst architectures, which will be addressed later in this article. Nevertheless, Hou et al. have optimized CsPbBr_3_ quantum dot (QD) sizes for CO_2_ reduction and found that 8.5 nm size leads to lower charge recombination, reaching 20.9 μmol g^–1^ h^–1^.^23^ Other approaches have been taken to develop photocatalysts, Xu et al. have supported CsPbBr_3_ on graphene oxide (GO) to reduce CO_2_, using it as a charge transport layer, permitting better charge separation and achieving 23.7 μmol g^–1^ h^–1^. Using GO also moved the selectivity slightly from CO to CH_4_ as a product (35% to 33% on an electron basis). The advantage of GO is illustrated in Figure 4a, where electrons are drawn out of the photocatalyst.^24^ The authors claim that GO separates the photoinduced charges because it draws electrons from the perovskite crystal CB edge. Kumar et al. have also shown that the addition of Cu NPs to a transport layer like reduced GO (rGO) can further increase production and selectivity, with a CsPbBr_3_-rGO-Cu catalyst reaching 90% selectivity toward CH_4_^21^ (Figure 4b)

### 2.3 Organic Degradation

Organic degradation is a common use of photocatalytic materials, especially as part of a water purification process, and titania, with its good activity and high stability, tends to be a common choice. HPs, on the other hand, find less use due to their instability in water. The reactions that describe the organic degradation process mostly involve radical formation and subsequent reactions with the organic at hand. The most important reactions are given in eqs 23–25, where the superoxide and hydroxyl radicals do most of the decomposition:
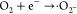
23
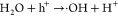
24
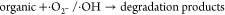
25

**Figure 5:**
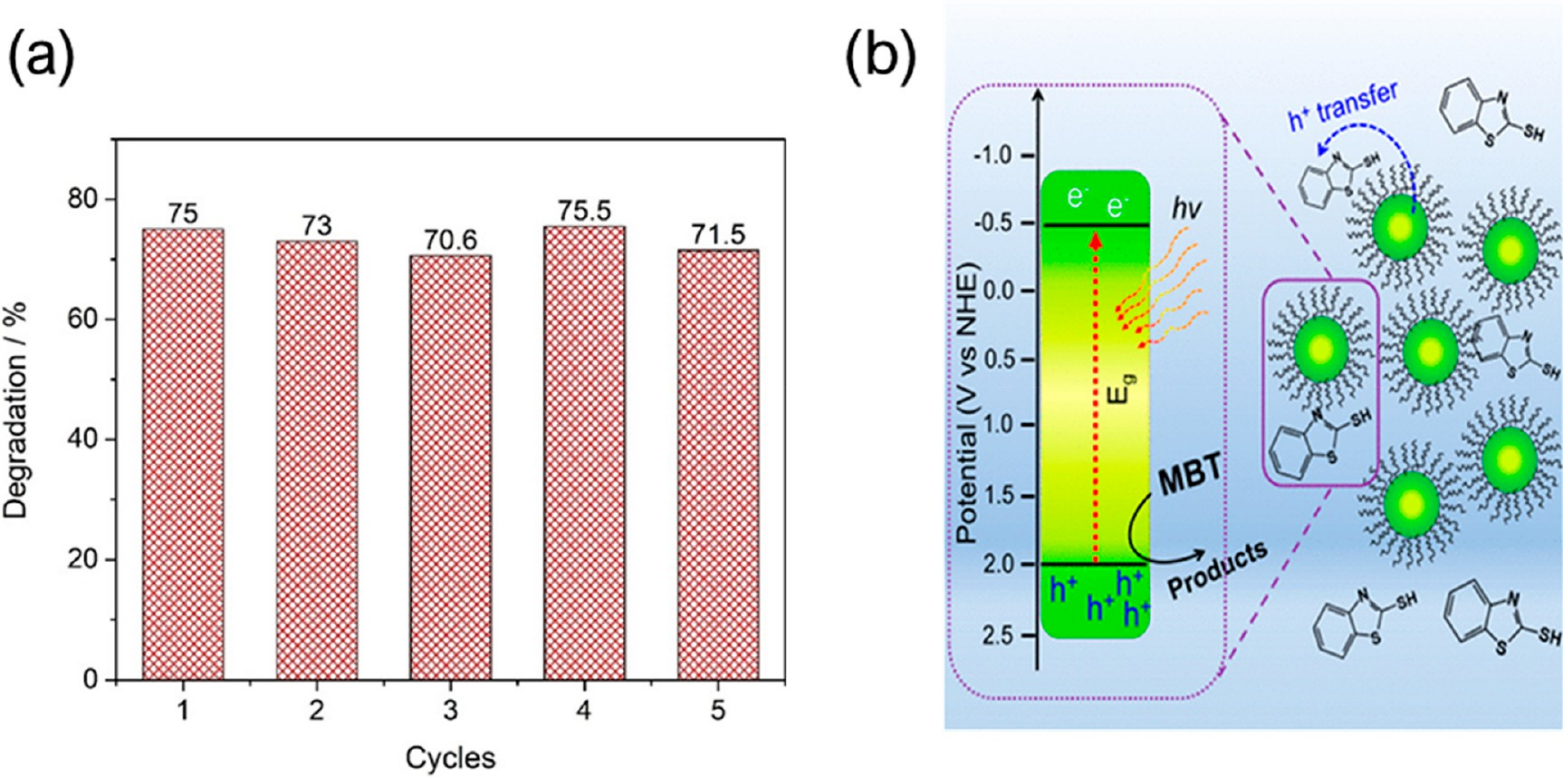
(a) Recyclability of CsSnBr_3_ for the degradation of crystal violet. Reproduced with permission from ref (26). Copyright 2018, John Wiley and Sons. (b) Scheme showing the direct oxidation of MBT, which does not rely on standard water-derived radicals, as the reaction is conducted in hexane. Reproduced from ref (27). Copyright 2019, American Chemical Society.

HPs themselves were used first for photocatalytic degradation in 2017 by Gao et al. for decomposition of methyl orange, through the photocatalytic formation of hydroxyl and superoxide radicals.^25^ They showed that CsPbCl_3_ outperformed TiO_2_ and ZnO for the decomposition of methyl orange in water. A tin halide perovskite, CsSnBr_3_ has been used to degrade crystal violet dye in water, again through the formation of superoxide and hydroxyl radicals, reaching a final degradation figure of 73.1%, with no significant decrease in performance after 5 cycles (Figure 5a).^26^ Through a different mechanism, CsPbBr_3_ was used to directly oxidize 2-mercaptobenzothiazole (MBT) in hexane (Figure 5b), with adsorption of the reactant directly on the catalyst under a reaction mechanism represented in eq 26, showing HPs also have the potential to oxidize organic compounds directly.^27^ Though the materials described in this paragraph are quite different, this further emphasizes how versatile HPs have become over the past decade of research, both in their use and their nature:
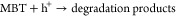
26

A silver–bismuth double perovskite, Cs_2_AgBiBr_6_, has been used to successfully degrade four different dyes, methyl orange, methyl red, and rhodamines B and 110, showing potential for a wide range of applications. It also dispenses with Pb, which could hinder the uptake of HPs for dye degradation in applications related to human consumption, such as water purification.^28^

### 2.4 Synthesis and Polymerization

One of the most interesting new emerging applications of HPs is its use in direct synthesis and polymerization reactions. If solar energy can be harnessed to directly facilitate the formation of C–C, C–O, and C–N bonds, it could significantly decrease the carbon footprint of the chemical industry.

**Figure 6:**
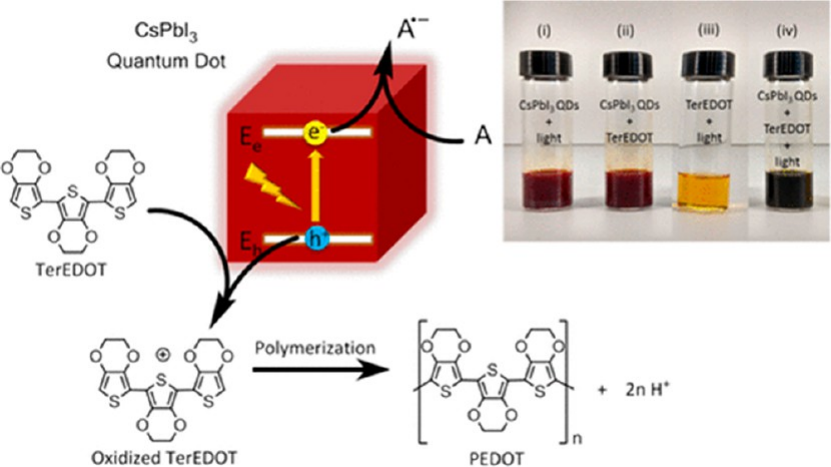
Direct polymerization of 3,4-ethylenedioxythiophene through oxidation, leading to encapsulation of CsPbI_3_ QDs with increased stability. Reproduced from ref (32). Copyright 2017, American Chemical Society.

Zhu et al. have shown that CsPbBr_3_ can catalyze the α-alkylation of aldehydes, promoting the formation of the C–C sp^3^ bond.^29^ Further, they demonstrated that this could be achieved under different experimental conditions, with different solvents. They also found that by tuning reactive conditions, they could alter selectivity for α-alkylation, sp^3^ C-coupling or even reductive dehalogenation, laying the groundwork for further organic synthesis. The mechanism for this was studied by Wang et al., who found that the reaction occurs through two intermediate ·C radicals which can then couple to form C–C bonds.^30^ Another approach has been to use the photogenerated charges instead of standard reductants or oxidizers, which is best exemplified by the work of Huang et al., who successfully oxidized benzylic alcohols into aldehydes on a TiO_2_/FAPbBr_3_ catalyst, achieving conversions of 63%.^31^ Further, CsPbI_3_ has been used to catalyze directly the polymerization of 3,4-ethylenedioxythiophene into its polymerized form, PEDOT, illustrated in Figure 6. Chen et al. have used this polymerization to directly encapsulate perovskite QDs, leading to increased stability, which shows the potential of using the photocatalytic ability of HPs as a key part of their design and preparation.^32^

The previous examples illustrate the diverse range of applications of HPs; however, the focus of this review will be primarily on photocatalytic CO_2_ reduction and H_2_ evolution. This is because in the area of dye degradation, a mature field already, HPs are outshone by more established and stable photocatalysts which can treat water without decomposing or adding extra risk with Pb^2+^. Organic synthesis is relatively new, and too broad in its application to generalize and benchmark operational conditions and figures of merit. We have as an aim to establish benchmarks for the field as well as to highlight strategies for the development of the most efficient photocatalytic systems, which is best explored through the study of solar fuel production, namely photocatalytic CO_2_ reduction and H_2_ evolution.

## 3 Halide Perovskite (HP) Photocatalysts and Developments

HPs have been well-known for at least a century but only in the 1990s they started to attract the interest of the scientific community. At the beginning of the 21st century, due to impressive optical and electronic properties, HPs were first researched for light-emitting devices and transistors.^33,34^ Then, in 2012, they started to be researched as sensitizing dyes and light absorption layers in solar cells, showing an outstanding potential to achieve solar-to-electricity conversion efficiencies above 15% with HP layers as thin as 330 nm.^8,33,35,36^ HPs showed performance comparable to the best III–V thin-film solar cells due to superior absorption cross section as well as small effective electron and hole masses due to strong *s-p* antibonding coupling.^36^ These superior optoelectronic properties were the catalyst to launching the extensive research of HPs. Further exploration of HPs revealed several other outstanding properties and qualities such as tunable band gap, shallow defect states, and cost-effective synthesis, making them excellent candidates for electronic and optoelectronic devices, such as solar cells, light-emitting diodes, gas sensors, photodetectors, and lasers. Recently, HPs have also been investigated for photocatalysis. In 2016, Park et al.^14^ demonstrated for the first time solar-driven H_2_ evolution on HPs. They developed a strategy for photocatalytic HI splitting using MAPbI_3_. This work prompted widespread research and application of HPs in photocatalysis.

### 3.1 Structure and Properties

**Figure 7:**
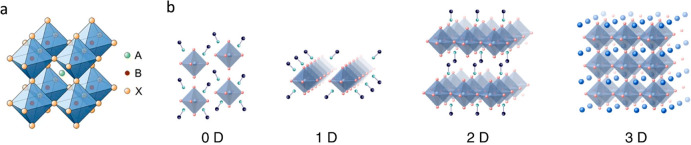
(a) Crystalline structure of ABX3 halide perovskites. Reproduced with permission from ref (43). Copyright 2017, Springer Nature. (b) Schematic representation of the different dimensionalities of HPs, from 0D to 3D ones. Reproduced with permission from ref (44). Copyright 2020, John Wiley and Sons.

Since 2016, a variety of HP materials have been produced that could find application in photocatalysis. All HP materials share a common crystalline structure ABX_3_ where A is a monovalent cation, B is a divalent cation and X is a halide ion (Figure 7a). Applying diverse synthesis strategies, a range of modified HP structures can be obtained. As a start, by engineering the A site, organic–inorganic perovskites can be produced, where A is an organic cation such as methylammonium (MA^+^, CH_3_NH_3_^+^) or formamidinium (FA^+^, (NH_2_)_2_CH^+^). For example, MAPbBr_3_ QDs with a size of 6 nm were prepared by hot injection of methylammonium bromide (MABr) and PbBr_2_ solutions into a preheated octadecene solution of oleic acid and octyl ammonium bromide.^37^ The obtained products had a blue shift of ∼16 nm in comparison with the bulk materials due to quantum size effects and a photoluminescence quantum yield (PLQY) of ∼20% as well as long stability for more than 3 months (dispersed in toluene). Moreover, the optical absorption properties and the photoluminescence (PL) properties of MAPbBr_3_ QDs can be tuned by varying the halide content, as it modifies the band gap energy.^38^

All-inorganic HP materials can be produced, where A is an inorganic cation such as cesium (Cs^+^) or rubidium (Rb^+^). Pioneering work on the preparation of all-inorganic HPs QDs was published by the Kovalenko group (discussed in more detail in Section 3.2.1).^39^ All-inorganic HPs are usually significantly more stable than organic–inorganic HPs with regards to temperature. Moreover, their optical properties can still be tuned by varying their halide content. Lead-free HPs, as its name suggests, avoid the use of toxic lead cations (Pb^2+^) and employ alternative cations such as tin (Sn^2+^), bismuth (Bi^2+^) or germanium (Ge^2+^) or cation pairs like silver (Ag^+^) and bismuth (Bi^+^) forming double HPs. The most studied examples of lead-free HPs are CsSnCl_3_ and FASnI_3_ for single HPs and Cs_2_AgBiX_6_ for double HPs.^28,40−42^ The substitution of Pb can result in nontoxic HPs but may lead to low stability and/or poor optoelectronic properties.

HPs can be obtained in various dimensionalities, such as 0D, 1D, 2D and 3D as a result of either different levels of corner sharing between MX_6_ octahedral anions or different crystal shapes. For example, 0D HPs show separated metal halide octahedral anions or metal halide clusters and 3D HPs show multiple corner-sharing MX_6_ octahedra (Figure 7b). Various low-dimensional HPs including 0D nanostructures (nanocrystals, quantum dots, and nanoparticles), 1D nanostructures (nanotubes, nanowires and nanorods) and 2D nanostructures (nanosheets and thin-films) have been reported in literature. Such a dimensionality change from 3D to low-dimensional structures leads to different optical and electronic properties such as band gap, density of states (DOS), and luminescence. A well-known example is CH_3_NH_3_PbBr_3_ NPs, where the change from bulk 3D crystals to 0D quantum dots results in a blue shift of the maximum of light absorption from 546 to 527 nm, due to particle-size quantum confinement.^37^

Due to their superior physical, chemical and optoelectronic properties such as tunable band gap, long charge-transport diffusion, high light absorption coefficients, and good defect tolerance, HPs have been considered promising candidates for photocatalytic applications.^45,46^ At the moment, HPs have been utilized in several photocatalytic applications such as CO_2_ reduction, H_2_O splitting, dye degradation, and organic synthesis.^18,32,47^ The universal features of HPs are determined by many parameters such as size, dimensionality (0D, 1D, 2D, and 3D), and chemical composition. The morphology, optical and electronic properties of HPs can easily be finely adjusted by controlling the reaction conditions such as choice of precursors, concentration, reaction temperature, and time, as we will discuss in the following section.

### 3.2 Preparation Methods and Photocatalytic Properties

The preparation of HPs is mainly carried out using bottom-up approaches, following either solution, gas phase or mechanochemical methods. The most studied solution-based methods are the hot-injection and ligand-assisted reprecipitation. For gas-phase methods, the main approach is chemical vapor deposition (CVD). Mechanochemical synthesis is a novel approach for HP preparation, where high-energy ball milling directly transfers kinetic energy through grinding balls to precursors which facilitates chemical reaction.

#### 3.2.1 Bottom-up Methods

**Figure 8:**
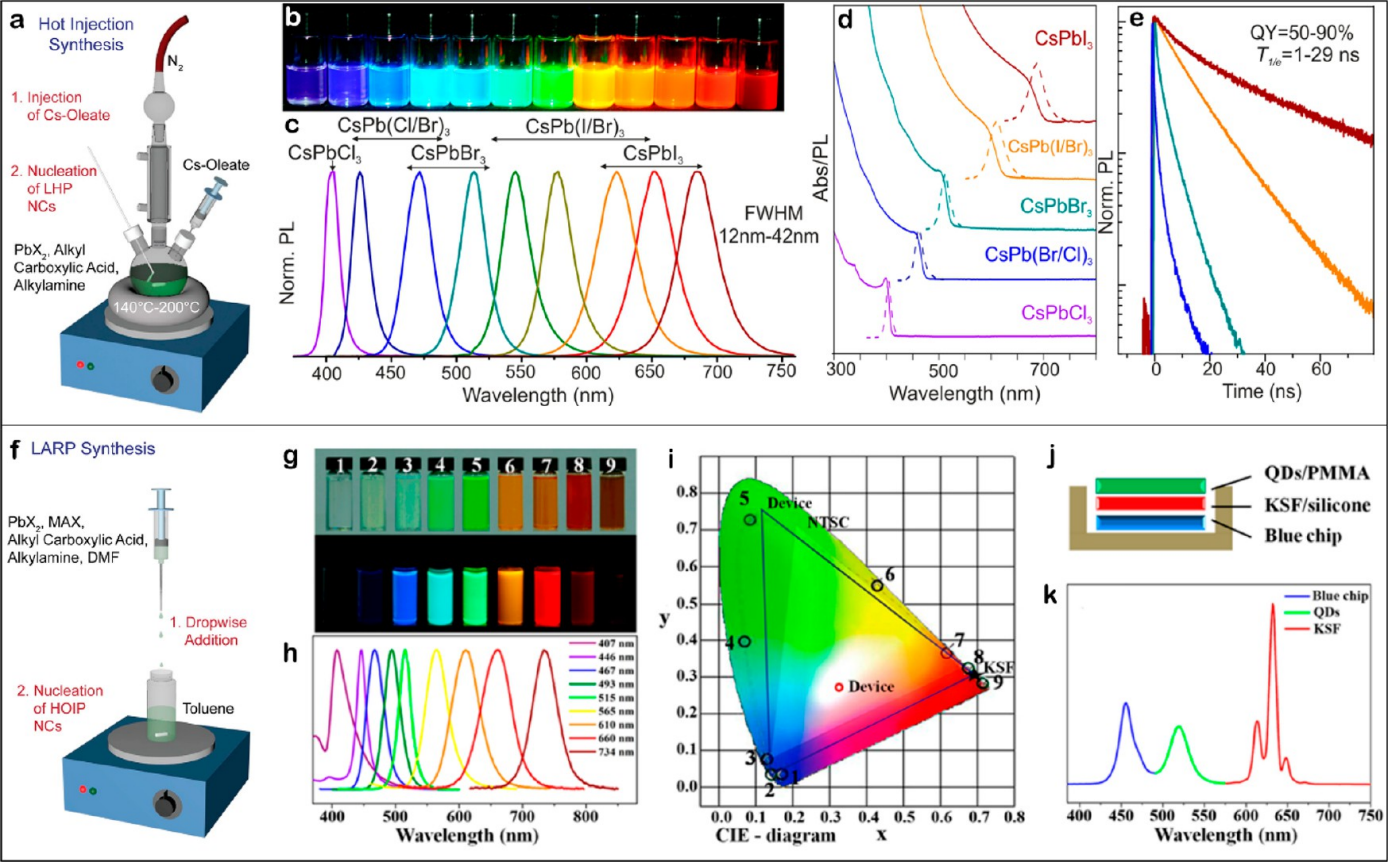
Schematic representation of (a) the hot-injection and (f) ligand-assisted reprecipitation (LARP) methods. Reproduced with permission from ref (54). Copyright 2021, John Wiley and Sons. Colloidal perovskite CsPbX_3_ NCs (X = Cl, Br, I) show size- and composition-tunable bandgap energies in the entire visible spectral region with narrow and bright emission: (b) optical images of colloidal solutions in toluene under UV irradiation (λ = 365 nm); (c) PL spectra upon excitation of λ_exc_ = 400 or 350 nm for CsPbCl_3_; (d) optical absorption and PL spectra; (e) time-resolved PL decays for all samples in (c) except CsPbCl_3_. Reproduced fromref (39). Copyright 2015, American Chemical Society; (g) optical images of MAPbX_3_ QDs under ambient light and UV lamp (λ = 365 nm); (h) PL emission spectra of MAPbX_3_ QDs; (i) CIE color coordinates corresponding to the MAPbX_3_ QDs (1–9, black circle), pc-WLED devices (blue lines), and NTSC standard (bright area); (j, k) schematic diagram and EL spectra of pc-WLED devices using green emissive MAPbBr_3_ QDs and red emissive rare earth phosphor KSF. Reproduced from ref (38). Copyright 2015, American Chemical Society.

Solution-based methods have been a preferred method for the fabrication of well-defined colloidal HP nanocrystals (NCs) in a controllable way. This approach can be used to produce high-quality and well-defined morphologies, such as 0D QDs, 1D nanowires, or 2D nanosheets.^48^ Since the publication of the hot-injection method by the Kovalenko group, this is one of the most applied methods for synthesis of all-inorganic HP QDs.^39^ For example, in a two-step approach, monodispersed colloidal CsPbX_3_ NCs (4–15 nm) were synthesized. First, a cesium oleate solution was obtained by decarbonization and drying of Cs_2_CO_3_ with oleic acid in 1-octadecene at 150 °C. This was followed by injection of PbX_2_ solution in 1-octadecene with oleic acid and oleylamine at 140–200 °C. Upon injection, HP QDs were formed immediately, and the reaction mixture was cooled down after 5 s (Figure 8a–e).

Metal halide salts are often used as a source of both cations and anions which offers limitations to control stoichiometry. To overcome this issue, Liu et al. designed the “three-precursor” hot-injection approach for the synthesis of CsPbX_3_ (X = Cl, Br, or I) NCs.^49^ The novelty was that instead of conventional PbX_2_ (X = Cl, Br, or I) salts, separate NH_4_X (X = Cl, Br, or I) and PbO were utilized as halide and lead sources, respectively. Guo et al. went further and elaborated a new strategy to enhance CO_2_ photocatalytic reduction, by modifying the halide ratio in the CsPb(Br_*x*_Cl_1–*x*_)_3_ structure.^50^ CsPb(Br_*x*_Cl_1–*x*_)_3_ were prepared by hot injection with different Cl/Br molar ratios. The photocatalytic activity results of the CsPb(Br_*x*_Cl_1–*x*_)_3_ (where *x* = 1, 0.7, 0.5, 0.3, 0) revealed that CO and CH_4_ production yields are notably influenced by the Cl/Br molar ratios in the structure. It was experimentally demonstrated that the photocatalytic activity of CsPb(Br_0.5_Cl_0.5_)_3_ was the highest, and the total amount of generated products in a 9 h experiment was 875 μmol g^–1^ with a high selectivity of 99% for CO and CH_4_.

Although the desired ion stoichiometry can be achieved using the “three precursors” method, their reactivity under these reaction conditions and method is low. To overcome this, a novel hot-injection method was developed by Creutz et al. They injected trimethylsilyl halides into a solution of metal acetate precursors (i.e., silver acetate, cesium acetate, and bismuth acetate) at 140 °C. The solution was then dissolved in 1-octadecene, oleic acid and oleyamine, which triggered immediate nucleation and growth of NCs.^51^ Wang et al. demonstrated that organic–inorganic hybrid perovskite methylammonium lead bromide (MAPbBr_3_) NCs can also be stabilized in aqueous HBr solution photocatalytically producing H_2_ under visible light. Moreover, they combined MAPbBr_3_ NCs with Pt/Ta_2_O_5_ and poly(3,4-ethylenedioxythiophene) polystyrenesulfonate NPs, which improved photocatalytic activity. The produced heterostructure demonstrated a 52-fold increase of H_2_ evolution compared with pristine MAPbBr_3_.^52^

In addition to the hot-injection method, ligand-assisted reprecipitation (LARP) technique is often used. The basic principle of this method is the dissolution of selected ions in a solvent until an equilibrium concentration is reached, followed by transfer of the solution to a nonequilibrium supersaturation state. The process is called ligand-assisted reprecipitation method if it involves stabilization ligands. This method proved its effectiveness by Zhang et al. in 2015 in the synthesis of organic–inorganic perovskite NCs. They prepared a precursor solution by dissolving PbBr_2_ and MABr salts in alkyl amines, carboxylic acids, and dimethylformamide. This solution was then dropped into toluene under vigorous stirring resulting in the formation of colloidal MAPbBr_3_ NCs, which had a PLQY around 70% at room temperature (Figure 8 f-k).^38^ Moreover, Dai et al. combined traditional ligand-assisted reprecipitation synthesis with spray pyrolysis method to produce cubic-shaped MAPbBr_3_ NCs with narrow size distribution around 14 nm.^53^ Specifically, MABr, PbBr_2_, oleic acid, octylamine, and dimethylformamide were sprayed into toluene, providing a large contact surface area between two solutions and homogeneous mixing, which led to the formation of well-defined cubic-shaped MAPbBr_3_ NCs.

To summarize, hot injection and LARP techniques are key methods for the preparation of perovskite NCs and QDs. The hot-injection method usually requires the absence of air and moisture and need to be conducted at elevated temperatures (up to 200 °C) allowing a more precise control of NC morphology compared to the LARP method. On the other hand, LARP is performed at lower temperatures and does not require specific equipment and processing conditions. However, due to the presence of polar solvents, stability of the as-prepared NCs is relatively low.

In addition to solution-based methods, HPs can be synthesized by gas-phase methods such as CVD. This method offers numerous benefits such as uniformity, scaling, and control of thickness. In 2014, Ha et al. synthesized the first organic–inorganic lead HP nanoplatelets by CVD.^55^ First, PbX (X = halide) plates were synthesized on top of muscovite mica by van der Waals epitaxial growth. Then, by thermally intercalating methylammonium halides, PbX was converted to MAPbX_3_ HPs. The size of the obtained MAPbX_3_ was in the range 5–30 μm with an electron diffusion length exceeding 200 nm. Moreover, Zhang et al. prepared CsPbBr_3_ and CsPbBr_*x*_Cl_3-x_ hemispheres with smooth surface and regular geometry on a silicon substrate by CVD. It was observed that surface morphology changed with the Cl/Br ratio. With increasing Cl content, the surface of CsPbX_3_ hemispheres became rougher due to vacancy defects. The HPs with hemispherical geometry provided an ideal platform for lasing and polarization development.^56^

A new milestone appeared in 2018 when the Kovalenko group prepared HPs by wet ball milling. As starting materials, they first prepared and used CsPbBr_3_ or FAPbBr_3_. Moreover, they showed that CsBr or FABr and PbBr_2_ precursors can be utilized for perovskite formation via wet ball milling. A zirconia bowl with 23 zirconia balls was loaded with HP bulk crystals or a mixture of powder precursors and mesitylene as a solvent and oleylammonium halide as a ligand. The obtained products were analyzed by transmission electron microscopy (TEM) revealing HP QDs with narrow size distribution and bright green luminescence under UV light.^57^

Recent findings by our (the Eslava) group shed light on the importance of choosing the right ball-milling parameters to control the resulting HP morphologies. Kumar et al. prepared CsPbBr_3_ of different morphologies such as nanorods, nanospheres and nanosheets, by simply choosing different milling times, zirconia ball sizes, and Cs precursors in a planetary ball mill. For example, using CsOAc as a Cs precursor resulted in nanorods of different aspect ratio depending on the milling time and ball size, but CsBr resulted in nanosheets. All these morphologies showed orthorhombic CsPbBr_3_ phase and photoluminescence emission at 530 nm upon 365 nm excitation.^21^ Kumar et al. also demonstrated the mechanochemical synthesis of double-perovskite Cs_2_AgBiBr_6_ nanosheets in a planetary ball mill using as precursors all the bromide cations.^58^ Both CsPbBr_3_ and Cs_2_AgBiBr_6_ nanosheets were tested for photocatalytic conversion of CO_2_ and H_2_O vapors under 1 sun of simulated sunlight, producing a mixture of CO, CH_4_, H_2_, and O_2_, showing a higher selectivity for CO production (reaching 1.5–2.3 μmol CO g^–1^ h^–1^).

Furthermore, the Biswas group demonstrated mechanochemical synthesis of halide perovskites with different dimensionalities: 3D CsPbBr_3_, 2D CsPb_2_Br_5_, 0D Cs_4_PbBr_6_, 3D CsPbCl_3_, 2D CsPb_2_Cl_5_, 0D Cs_4_PbCl_6_, 3D CsPbI_3_, and 3D RbPbI_3_.^59^ They showed the importance on the choice of precursors for desired stoichiometric ratios and the possibilities on postsynthetic structural transformations to different dimensionalities. This group also demonstrated mechanochemical synthesis of Pb-free Ruddlesden–Popper-type layered Rb_2_CuCl_2_Br_2_ perovskite using a few drops of dimethylformamide solvent as a liquid assistant, achieving a band gap around 1.88 eV and a band-edge PL emission at 1.97 eV at room temperature.^60^ In another work, they demonstrated the mechanochemical synthesis of novel Pb-free layered RbSn_2_Br_5_. By tuning the halide ratio, the 3.20 eV band gap of RbSn_2_Br_5_ changed to 2.68 eV (RbSn_2_Br_4_I) and 3.36 eV (RbSn_2_Br_3_Cl_2_). The layered RbSn_2_Br_5_ showed thermal stability up to 205 °C while maintaining crystalline stability at ambient conditions for 30 days. The optical properties and charge-carrier recombination dynamics were further studied. It was confirmed that recombination at 298 and 77 K takes place through the band-edge and shallow states with average lifetimes in the range of few nanoseconds.^61^

#### 3.2.2 Halide Perovskites-Based Composites

Pristine HPs are highly sensitive to moisture, light, and temperature owing to their low formation energy. Their instability limits their wider utilization in photocatalysis. Therefore, one of the strategies employed to overcome these drawbacks and further improve optical and charge-separation properties is the preparation of HP-based heterostructures. Raja et al. demonstrated the preparation of HP-based nanocomposites by embedding CsPbBr_3_ nanocubes, nanoplates, and nanowires in hydrophobic macroscale polymeric matrices such as polystyrene. HPs were prepared by the conventional hot-injection method and further blended with the polystyrene in toluene. After mixing and sonicating, the solution was spin-coated onto coverslips. It was stated that because of the high viscosity it was challenging to obtain uniform thin-film morphology. The PLQY of the HPs based nanocomposite remained stable during 4 months of immersion in water. Moreover, the photostability of the as-prepared nanocomposite was improved upon encapsulation, achieving 10^10^ absorbed photons per QD at 10^5^ W cm^–2^ excitation flux.^62^

Schuenemann et al. prepared a CsPbBr_3_/TiO_2_ nanocomposite by a low-temperature wet impregnation method. TiO_2_ (P25) was thoroughly mixed with a dimethyl sulfoxide solution containing CsPbBr_3_ precursors. The resultant substance was dried for 4 h at 60 °C to evaporate the dimethyl sulfoxide and crystallize CsPbBr_3_. The prepared nanocomposite demonstrated enhanced visible-light selective photocatalytic oxidation of benzylalcohol toward benzaldehyde as well as good morphological stability and crystal structure.^63^ Chen et al. demonstrated a novel strategy for improving the photocatalytic reduction of CO_2_ by immobilizing Ni metal complex Ni(tpy) on the surface of CsPbBr_3_ by electrostatic interaction. CsPbBr_3_ was prepared by the traditional hot-injection method, followed by substitution of surface organic ligands for PF_6_^–^. The Ni metal complex further electrostatically interacted with PF_6_^–^ on the HP surface, producing a stable hybrid structure CsPbBr_3_–Ni(tpy). The Ni metal complex immobilization on CsPbBr_3_ was reported to be critical for highly efficient CO_2_ reduction, because it was assigned to work as an electron sink, rapidly accepting photoexcited electrons from the CB of CsPbBr_3_. The photocatalytic reduction of CO_2_ by CsPbBr_3_–Ni(tpy) exhibited 1724 μmol g^–1^ of CO/CH_4_, which was almost 26 times higher than that of pristine CsPbBr_3_.^64^

Mechanochemical synthesis approaches have also been developed for HP composites. Kumar el al. demonstrated the successful preparation of composites of CsPbBr_3_ nanosheets and Cu-loaded reduced graphene oxide (Cu-rGO) following a solvent-assisted mechanochemical synthesis in a planetary ball mill that ensured proper mixing of reactants in the presence of large rGO flakes (1–100 μm).^21^ TEM characterization demonstrated the growth of HP on Cu-rGO, moreover showing templating effects and intimate contact between phases. The same approach was also demonstrated for double-perovskite Cs_2_AgBiBr_6_ composites with Cu-rGO.^58^ TEM characterization also demonstrated intimate contact between phases and X-ray photoelectron spectroscopy presented features assigned to Ag–O and Bi–O bonding between the HP and rGO. The formation of HP composites with Cu-rGO drastically boosted the photocatalytic conversion of CO_2_ and H_2_O vapors under 1 sun of simulated sunlight. For example, CsPbBr_3_–Cu-rGO composites achieved 12.7(±0.95) μmol CH_4_ g^–1^ h^–1^, 0.46(±0.11) μmol CO g^–1^ h^–1^, and 0.27(±0.02) μmol H_2_ g^–1^ h^–1^. Importantly, the presence of Cu-rGO enhanced the hydrophobic character of the HP-based composite and ensured charge separation. These effects boosted the reusability of the composite for photocatalytic reactions, so 90% of their photocatalytic production rate was retained over three consecutive cycles.

#### 3.2.3 Halide Double Perovskites and Halide Perovskite Derivatives

As mentioned in Section 3.2.2, HPs are known for their sensitivity to light, moisture, and temperature. In addition to that, the vast majority of HPs studied in literature contain Pb in the B-site. Pb^2+^ is considered a source of toxicity and an environmental hazard.^65−67^ Recently, scientists have developed new approaches to tackle the aforementioned challenges. These approaches include, but are not limited to, the reduction of crystal defects introduced during synthesis, structural doping, and the use of efficient charge transport layers to reduce charge recombination.^68,69^ Even though these methods lead to better stability, the challenges related to the presence of Pb in the structure are not solved. Attempts to replace the divalent Pb cation with Sn^2+^ or Ge^2+^ have been conducted. However, the 5*s* orbitals of these candidates can further decrease the material stability.^70^

Recently, the idea of synthesizing halide double perovskites has been explored as a green alternative to lead-based perovskites. The process entails designing materials where two Pb atoms are replaced either with one monovalent and one trivalent cation (A_2_B^+^B^3+^X_6_) or with one tetravalent cation (A_2_B^4+^□X_6_) and a vacancy (□). This method further broadens the list of potential cations to replace lead. For example, Cs_2_AgBiBr_6_ nanocrystals have demonstrated remarkable stability at 100, during prolonged illumination, and in humid conditions. Its stability, suitable band structure, good light absorption properties, and long carrier recombination lifetime allowed its use as a photocatalyst for CO_2_ reduction reactions.^58,71^ Computationally, researchers have also identified structures like Cs_2_NaBiCl_6_, Cs_2_AgInCl_6_, (MA)_2_AgBiBr_6_, and Cs_2_AgBiCl_6_ as potential photocatalysts.^72−74^

**Figure 9:**
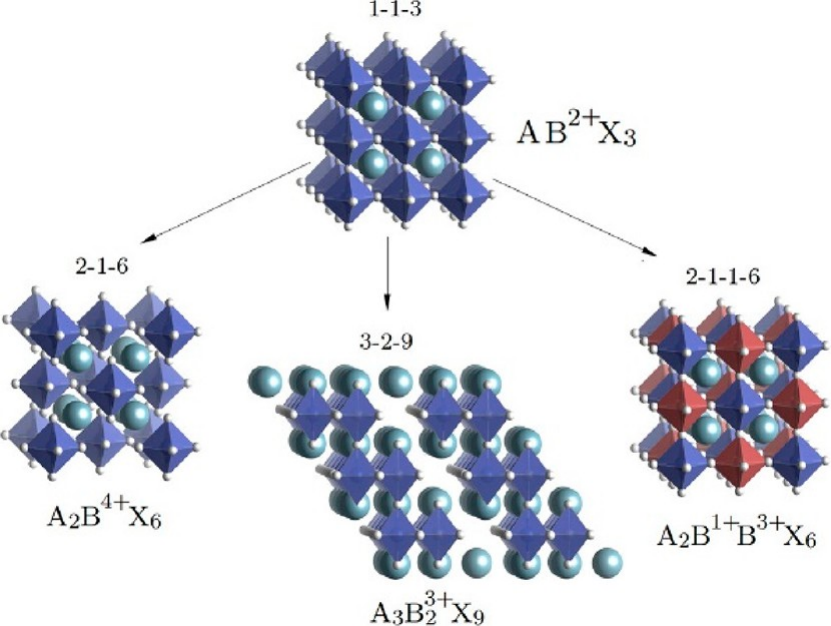
Schematic representation of a perovskite crystal (1–1–3) and its derivatives after replacing two divalent B-site cations with (2–1–6) a tetravalent cation, (3–2–9) a trivalent cation, and (2–1–1–6) a tri- and monovalent cation. The string of digits above the structures refers to the vacancy order of the ions, respectively. Reproduced from ref (78). Copyright 2015, American Chemical Society.

In addition to double perovskites, researchers have identified other possible lower dimensional halide perovskite derivatives. An example would be of the form A_3_X_9_ as demonstrated in Figure 9. Different atomic combinations have already been synthesized and tested including Cs_3_Sb_2_I_9_, Cs_3_Bi_2_I_9_, MA_3_Bi_2_I_9_, and Rb_3_Sb_2_I_9_. For example, Cs_3_Bi_2_I_9_ has shown good light absorption in the visible range with a bandgap of 1.9–2.2 eV depending on the synthesis route. It was also found to have good thermal stability up to 425 as well as light stability to around 120 days.^75,76^ Bhosale et al. performed a comparative study between three bismuth iodide perovskites having Cs, Rb, and MA in the A-site. The group synthesized the three perovskites using an ultrasonication method and deduced that Cs_3_Bi_2_I_9_ showed a significantly higher yield of CO than its counterparts due to superior charge transfer.^77^

## 4 Key Factors Influencing the Performance

Several material-related factors highly influence the photocatalytic performance of HP photocatalysts such as stability, band structure, morphology, size, charge separation, adsorption capacity, and selectivity. In this section, we will have a closer look at each of those and highlight its role in the photocatalytic process.

### 4.1 Stability

Stability has an essential role in liquid and gas-phase photocatalysis involving HPs. Although all-inorganic HPs have higher thermal stability than organometallic structures, their utilization is still a challenge due to their highly polar ionic structure. Polar solvents such as water are harmful to HPs, and in order to maintain stability and enhance photocatalytic performance, several approaches have been developed. One of the most promising methods to enhance the stability of HPs is encapsulation, which, in addition to stability, can improve photocatalytic performance. Encapsulation of HP structures was first demonstrated with polymer materials, providing hydrophobic protection. The most common polymers which were used for the protection of HPs are polystyrene and poly(methyl methacrylate).^79−81^

In 2016, Wang et al. engineered a monolithic superhydrophobic polystyrene fiber membrane with CsPbBr_3_ QDs by a one-step electrospinning process. The as-prepared composite structure exhibited high quantum yields (∼91%) and kept nearly 80% fluorescence after 365 nm UV-light (1 mW cm^–2^) illumination for 60 h.^82^ Further, Ma et al. embedded CsPbBr_3_ in polystyrene and poly(methyl methacrylate) by an effluent-free microfluidic spinning technique, producing 1D–2D microreactors which continuously synthesized embedded composite material. The CsPbBr_3_/ polystyrene and poly(methyl methacrylate) nanocomposite demonstrated tunable emission between 450 and 625 nm and great PL stability against water vapors and UV radiation.^83^

**Figure 10:**
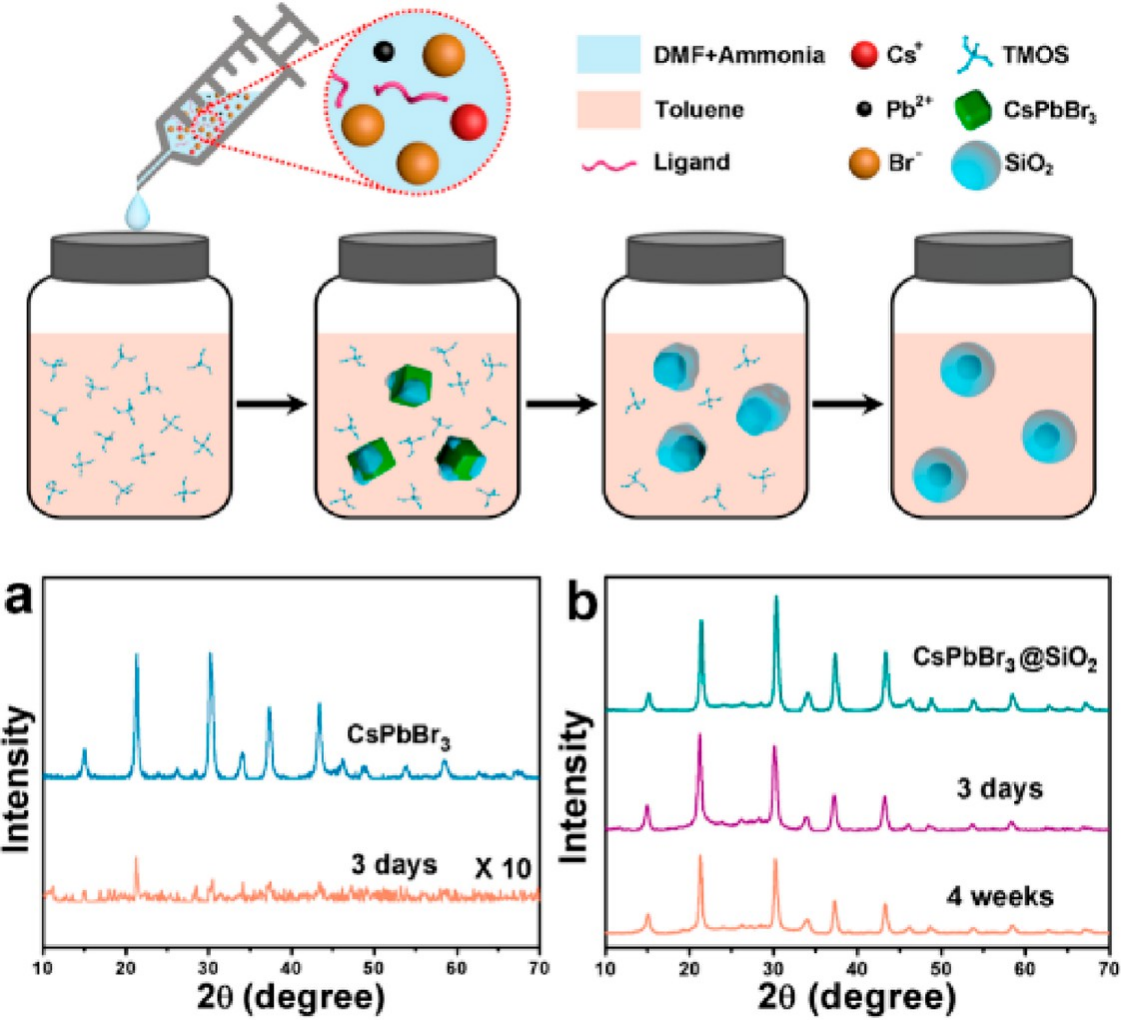
Proposed formation of CsPbBr_3_@SiO_2_ core–shell NPs, and XRD pattern highlighting stability of (a) CsPbBr_3_ NCs and (b) CsPbBr_3_@SiO_2_ core–shell NPs. Reproduced from ref (84). Copyright 2018, American Chemical Society.

The coverage of HP surfaces with various oxides (semiconductors and insulators) has also been studied. Zhong et al. prepared one-pot monodisperse CsPbBr_3_@SiO_2_ core–shell NPs. The generation of SiO_2_ oligomers led to the growth of a SiO_2_ shell around CsPbBr_3_ in the presence of ammonia and tetramethyl orthosilicate (Figure 10)_._ Various parameters such as reaction temperature, precursor species, pH value as well as CsPbBr_3_ and SiO_2_ concentrations determined the successful core–shell formation. As a result, CsPbBr_3_@SiO_2_ demonstrated higher long-term stability against water vapors and ultrasonication in comparison with pristine CsPbBr_3_ NCs. (Figure 10a,b)^84^ Impregnation of CsPbBr_3_ in TiO_2_ is considered extremely promising since TiO_2_ itself can facilitate photocatalytic activity by proper band alignment. Xu and co-workers coated CsPbBr_3_ NCs with amorphous TiO_2_. The TiO_2_ shell around HPs was able to suppress the intrinsic radiative recombination, providing facilitated transport of photoexcited electron–hole pairs as well as enhanced photocatalytic activity due to improved CO_2_ adsorption. A combination of the above-mentioned effects boosted photocatalytic activity up to 6.5 times and stability was extended up to 30 h.^85^

Carbon-based materials have been found to be beneficial for enhancing the stability of HPs. Ou et al. demonstrated the preparation of CsPbBr_3_ QDs on NH_*x*_-rich porous g-C_3_N_4_ nanosheets by a self-assembly method that forms the composite via N–Br interaction. The formation of N–Br bonds led to enhanced charge separation, longer lifetime of photoexcited electron–hole pairs, and provided an alternative way for surface passivation reasonably improving stability. The CsPbBr_3_ QDs/g-C_3_N_4_ nanocomposite exhibited enhanced photocatalytic activity toward reduction of CO_2_ to CO, which was 15 times higher than the activity of pure CsPbBr_3_ QDs. Moreover, the nanocomposite was endowed with outstanding stability in acetonitrile/water and ethyl acetate/water systems.^86^

Metal–organic frameworks (MOFs) have been similarly employed to improve and maintain the stability of HPs. Wu and co-workers managed to enhance the stability of MAPbI_3_ QDs by coating them with Fe-based MOF PCN-221[Fe_*x*_(*x* = 0–1)] following a sequential deposition route. The close contact between QDs and the Fe catalytic sites in the MOF enabled the swift transfer of photoexcited electrons, improving the photocatalytic CO_2_ reduction. Namely, the photocatalytic reduction of CO_2_ to CO and CH_4_ was 38 times higher than that of PCN-221(Fe_0.2_) alone, using H_2_O vapor as the electron source. Moreover, the stability was significantly improved—no detected changes in crystalline properties for over 80 h.^87^

In addition, HPs become unstable upon heating and/or UV irradiation. A reported approach to mitigate these issues is the preparation of HP-based nanocomposites with more thermally stable materials and/or photostable materials. Gao and co-workers produced CsPbBr_3_ QDs assembled into silicon oxide via a one-pot nonpolar solvent synthesis method at a nanoscale-particle level. CsPbBr_3_@SiO_2_ nanocomposites exhibited superior photostability for up to 168 h of UV irradiation as well as higher stability against moisture for up to 8 days, while maintaining a high PLQY of 87%.^88^ The relatively low formation energy was assigned to be responsible for the internal defect formation. Therefore, doping with divalent metal cations with a smaller radius than Pb may result in contraction of the M–X bond length and higher formation energy and hence stability. Zou et al. demonstrated doping of Mn^2+^ ions in CsPbX_3_ (X= Cl, Br, and I) QD lattices. The prepared QDs were thermally stable up to 200 °C temperature under ambient air conditions and maintained the PL intensity for three cycles.^89^

It is important to emphasize that a lot of the developed strategies to increase the stability of HPs, including some of the ones here detailed, such as encapsulation in insulating SiO_2_, work well in the field of light-emitting diode devices, where the goal is radiative recombination upon illumination. In photocatalysis, the charges need to be extracted and some of these approaches would severely hinder the charge extraction.

### 4.2 Electronic Band Structure

HPs are well-known for having a widely tunable band gap. The band gap energies of HPs range from UV to near-infrared wavelengths. Such broad band gap energy range is determined by various parameters including the halide composition, crystal size, and crystalline phase. The electronic band structure is an important feature when designing heterostructures, since adequate band alignment facilitates the separation of photoexcited electrons and holes and further promotes photocatalytic activity. HPs can be prepared in different dimensions such as 0D NCs or QDs, 1D nanowires, and 2D nanosheets. Smaller crystal dimensions can increase the band gap energy due to quantum confinement effects.

The nature of the band gap in HP materials was recently studied by first principle calculations. Yuan et al. analyzed various electronic structures of ABX_3_ perovskites where A = CH_3_NH_3_, Cs; B = Sn, Pb; X = Cl, Br, I by density functional theory (DFT) using the Perdew–Burke–Ernzerhof (PBE) exchange–correlation functional and the Heyd–Scuseria–Ernzerhof (HSE) hybrid functional. They confirmed that the valence band maximum (VBM) results from antibonding hybridization of B *s* and X *p* states and the conduction band minimum (CBM) from π antibonding of B *p* and X *p* states. It was further found that the contribution of the A site toward VBM or CBM is insignificant, but it affects the lattice constants that impacts band gap width. The CBM gradually decreases upon halide exchange according to the Cl → Br → I scheme, whereas the VBM depends on the atomic orbital energy of X *p* site and bond length of B–X. The robust antibonding in B*s*–X*p* state is considered as more delocalized than the X *p* alone, which results in smaller hole effective mass and larger hole mobility.^90^

The electronic structure of the semiconductor is determined by two important physical parameters: binding energy of the exciton (R*) and reduced effective mass (μ). Galkowski and co-workers explored electronic properties of methylammonium and formamidinium lead trihalide perovskite, namely MAPbI_3_, MAPbBr_3_, FAPbI_3_ and FAPbBr_3_ by means of magneto-optical absorption spectroscopy. They demonstrated that R* and μ parameters can be directly determined at low temperatures (2 K) by measuring excitonic states in the magnetic field and Landau levels of the free carrier states. The results of the work emphasized the efficiency of magneto-optical absorption spectroscopy toward determination of R* and μ parameters at low temperature. In addition, a relationship between R*, μ and material band gap was observed.^91^

The same method was used to determine R* and μ parameters in all-inorganic HPs. Baranowski and co-workers applied magneto-optical absorption spectroscopy to CsPbCl_3_ to identify them. A CsPbCl_3_ perovskite film was grown on a muscovite mica substrate using a CVD method. Applying low-temperature transmission spectroscopy in pulsed magnetic fields up to 68 T resulted in direct observation of R* and μ parameters. The observed values were in good agreement with experimental and calculated DFT values.^92^

Li et al. have observed enhanced multiple exciton generation efficiencies in intermediate-confined FAPbI_3_ NCs. They demonstrated higher multiple exciton generation efficiencies (up to 75%) and low- multiple exciton generation threshold energies to 2.25 eV by dimensional tuning of FAPbI_3_ NCs. It was calculated that band gap energies increased from 1.5 eV in the bulk to 1.7 eV for NCs with an edge length of ∼8 nm.^93^ Vashishtha et al. prepared thallium-based HPs Tl_3_PbX_5_ (X= Cl, Br, I). Varying colloidal methods, spheroidal NCs and nanowires were produced. It was shown that their band gap can be tuned by halide substitution and creating nanocomposites that strongly absorb in the range between 250 and 450 nm. Moreover, Tl_3_PbBr_5_ NCs exhibited a quantum confinement effect upon size tuning.^94^ Dou et al. developed a solution-phase growth strategy of large-size, square-shape (C_4_H_9_NH_3_)_2_PbBr_4_ single-crystalline nanosheets. These nanosheets revealed intriguing features such as structural relaxation and vivid PL compared to the bulk material. The band gap energies ranged from 2.97 eV for the bulk material to 3.01 eV for the nanosheets, indicating lattice expansion. Furthermore, by altering nanosheet thickness and composition, natural color tuning was achieved.^95^

Both compositional and dimensional engineering are promising approaches to modify the band gap energies of HPs. Tuning the halide (X) composition and developing mixed-halide perovskites is an accessible approach to obtain HPs of different band gap energies. For example, Akkerman et al. established a halide-reverse strategy for the synthesis of CsPbBr_3_ NCs along with anion exchange reactions. Preparing CsPbBr_3_ NCs by hot-injection method, they achieved NCs with a band gap of 2.43 eV. Upon halide substitution (Br to Cl), CsPbCl_3_ NCs exhibited a gradually increased band gap up to 3.03 eV. Further halide replacement to I resulted in the decreased band gap to 1.88 eV. The anion exchange process is considered a versatile technique for gaining novel structural and optical properties of CsPbX_3_ NCs (X = Cl, Br, I).^96^

**Figure 11:**
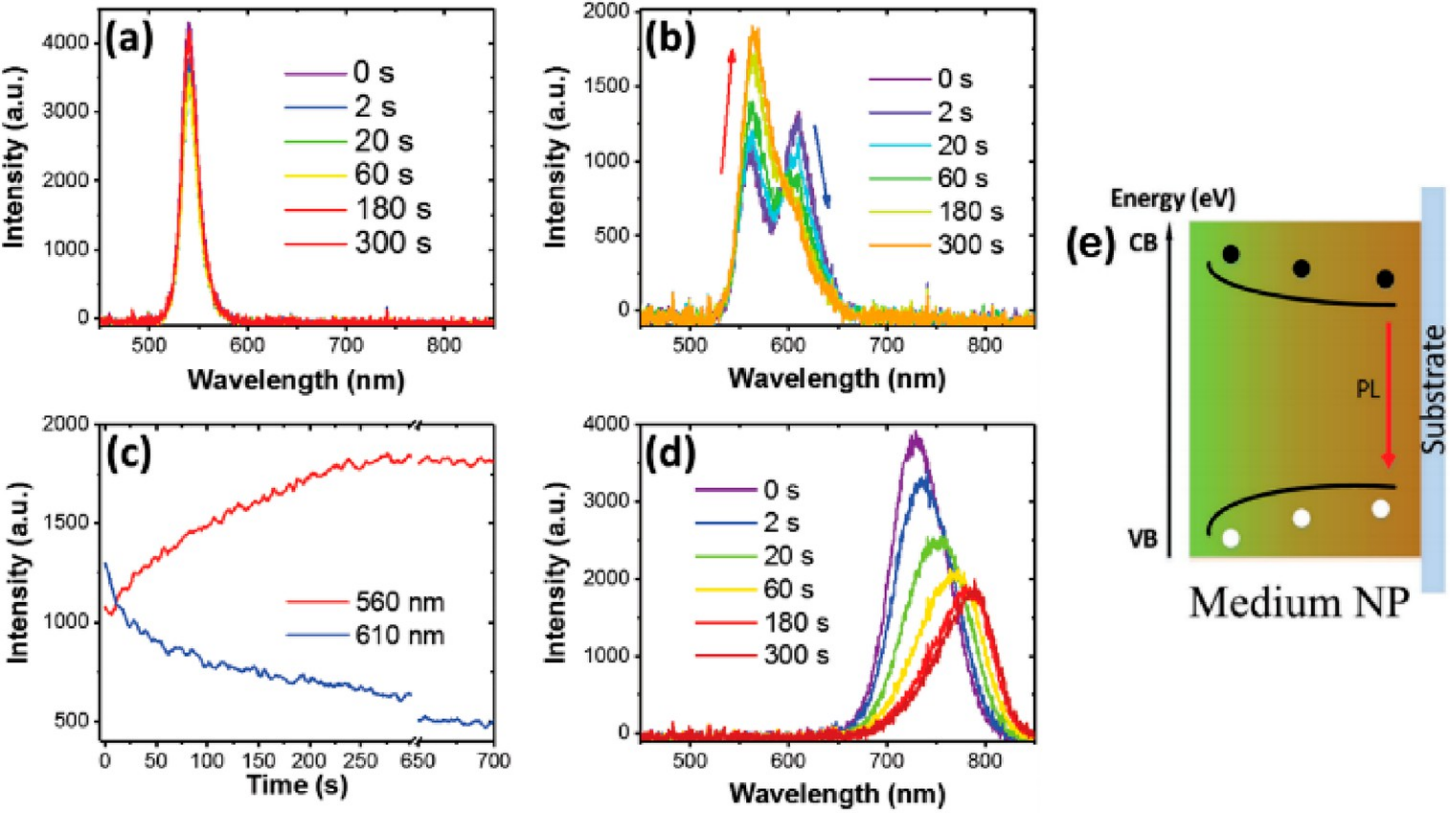
Specific gradient bandgap structure in FAPbX3. PL spectra under continuous illumination of materials with different thicknesses (a) 58 nm, (b) 239 nm, and (d) 1.3 μm (λ_exc_ = 405 nm). PL intensity of the two peaks of the medium thickness NP (c) as a function of the illumination time and (e) a schematic diagram of the gradient energy band structures. Reproduced with permission from ref (97). Copyright 2018, Wiley-VCH.

The halide migration-induced phase segregation is another efficient way to modify the band gap of HPs. In 2018, Zhou et al. demonstrated a two-step CVD preparation method of mixed-halide FAPb(Br_*x*_I_1–*x*_)_3_ NPs. First, FAPbI_3_ NPs were prepared, and then they were treated with FABr vapors. Upon FABr vapor treatment, Br^–^ replaced I^–^ from the Br-rich site on top to the I-rich site at the bottom. Once the substitutional process was completed, a specific gradient band gap structure appeared, ranging from 2.29 eV for FAPbBr_3_ to 1.56 eV for FAPbI_3_ (Figure 11). It was revealed that in such a gradient band gap structure, photogenerated carriers were shuttled to the low band gap edge by energy funneling effects.^97^

The band gap energies of HPs also strongly depend on the present phase. Usually, thermal effects are responsible for phase transitions, resulting in the reconstruction of the crystals to another space group (not always a HP phase). Studying the effects of the temperature on band gap energies and emission decay dynamics of MAPbI_3_, MAPbBr_3_, and FAPbBr_3_ using time-resolved PL spectroscopy, Dar and co-workers observed an extraordinary PL behavior. Two distinct emission peaks appeared in MAPbI_3_ and MAPbBr_3_ under low temperature (less than 100 K) while FAPbBr_3_ showed a single emission peak. A deeper analysis revealed an unusual blueshift of the band gap with increasing temperature from 15 to 150 K. The blueshift originated upon stabilization of the VB maximum and by the coexistence of MA-ordered and MA-disordered orthorhombic domains. The FAPbBr_3_ possessed only a single emission feature due to a low difference between ordered and disordered FA orthorhombic domains [∼10 to 20 meV in FAPbBr_3_ versus ∼80 to 90 meV in MA perovskites].^98^

### 4.3 Size and Morphology

Morphological and structural properties have a strong influence on the photocatalytic activity of HPs. HPs can be prepared as nanocrystals, nanoparticles, nanofibers, nanoplates, thin-films, and periodic structures (periodical lines, hierarchical structures, and patterned single crystals). 0D HPs possess high quantum yield, narrow-band emission, and defect-tolerant band gap and, thus, they have great potential for optoelectronic and photocatalytic applications. The existing preparation methods allow rapid fabrication of sophisticated 0D HPs with a large area in a cost-effective way.^23,99,100^ Moreover, 0D HPs can be coupled with other nanoscale materials leading to enhanced photocatalytic performances. Pioneering work on the preparation of 0D HPs was performed by Schmidt et al.^37^ MAPbBr_3_ QDs with an average size of 6 nm were fabricated using an ammonium bromide with organic ligand chains. It was shown that the stability of the as-prepared materials is maintained for more than three months. Further, as previously discussed, Kovalenko and co-workers also prepared all-inorganic CsPbBr_3_ QDs by hot-injection method. The morphology and size of QDs were tuned between 4 and 15 nm varying the reaction conditions such as reaction temperature and choice of halide precursors. The sizes and composition of CsPbBr_3_ QD strongly affected the PL properties of QDs.^39^

**Figure 12:**
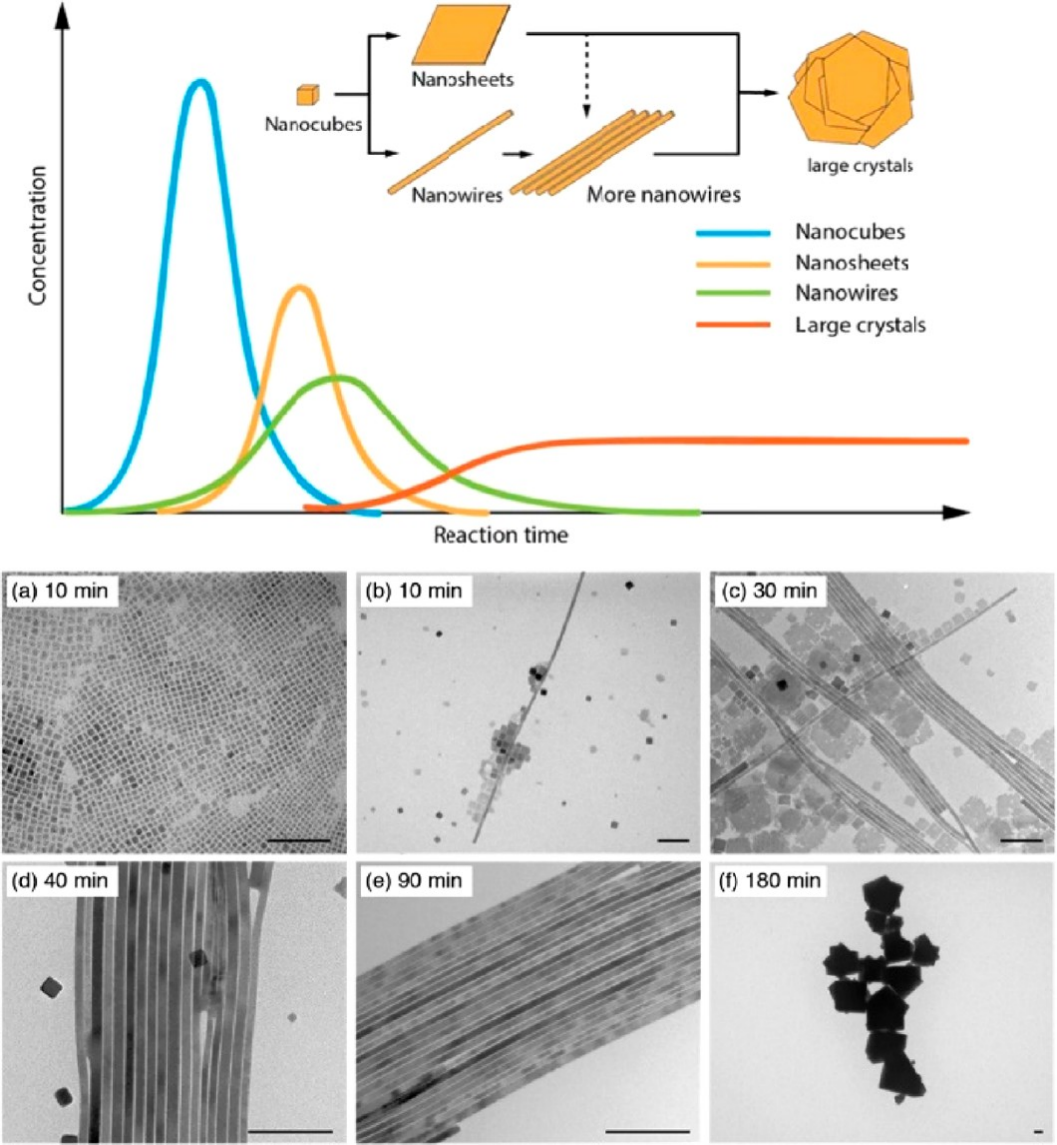
Sketch represents dependence between reaction time vs concentration of different morphological modifications and shape evolution of CsPbBr3 nanostructures during various reaction times (a, b) 10 min, (c) 30 min, (d) 40 min, (e) 90 min, and (f) 180 min. Scale bar is 100 nm. Reproduced from ref (48). Copyright 2015, American Chemical Society.

1D HPs have one microscopic and two nanoscopic dimensions. Furthermore, due to the intrinsic high aspect ratio, 1D HPs are endowed with a two-dimensional quantum confinement effect. The fabrication of 1D HPs and 0D HPs are alike, where different parameters such as temperature, time, mixing speed, and precursor concentration determine the growth of the nanostructures. Aharon et al. explored a low-temperature synthesis of HP nanorods. The HP nanorods demonstrated a narrow size distribution and a strong PL emission. It was shown that the shape and size of the nanorods were not affected by the halide ratios and the average dimensions were measured to be 2.25 ± 0.3 nm in width and 11.36 ± 2.4 nm in length. Moreover, the band gap energies were simply adjusted by halide composition ranging from 1.9 eV for iodide to 2.26 eV for bromide.^101^ The preparation of all-inorganic HPs nanowires was demonstrated by Zhang and co-workers through fabricating CsPbX_3_ (X = Br, I) nanowires using a catalyst-free, solution-phase synthetic method. The uniform growth of single-crystalline, orthorhombic CsPbX_3_ nanowires of approximately 12 nm in width and 5 μm in length was observed after 40–90 min of reaction (Figure 12). Further, analysis of optical properties showed that CsPbBr_3_ and CsPbI_3_ nanowires have PL and temperature-dependent PL. Additionally, CsPbI_3_ nanowires exhibited a self-trapping effect.^48^

Climbing up, 2D HPs have one nanoscopic and two microscopic dimensions. Upon precise tailoring of thickness and dimensions of these materials, unique properties such as superb light absorption and emission, optical transparency, and high specific surface area can be achieved. The fabrication of 2D HP materials can be achieved using relatively simple techniques such as spin coating and drop-casting. Levchuk and co-workers prepared quantum size confined MAPbX_3_ (X = Br and I) 2D nanoplates by LARP. This approach made it possible to tune the thickness of the nanoplates by varying the ratios of oleylamine and oleic acid ligands. A quantum confinement effect was observed, making band gap tuning more efficient.^102^ It was possible to achieve remarkable PLQYs of 90% for MAPbBr_3_ and 50% for MAPbI_3_ nanoplates. There are several other examples of all-inorganic 2D HPs. For example, the successful fabrication of a few-unit-cell-thick 2D CsPbBr_3_ nanoplates via a room-temperature ligand-mediated method was reported by Sun et al. They demonstrated the preparation of CsPbBr_3_ QDs, nanorods, nanocubes, and nanoplates by the reprecipitation approach, varying organic acid and amine ligand concentrations. The as-prepared 2D CsPbBr_3_ nanoplates had a typical length of around 100 nm and thickness of about 5.2 ± 1.3 nm. It was concluded that the PL decay lifetimes depend on the size, shape, and composition of the HP nanostructures.^103^

Preparation of HP-based 3D periodic structures improves interaction with the light, benefiting from the intrinsic properties of HPs as well as from the features of periodic structures. It is possible to improve the properties of HPs and expand their use in photocatalytic, optical and electronic applications by creating hierarchical and periodic structures. Jeong and co-workers focused on micropatterning of HP films. They upgraded solvent-assisted gel printing methods with high-boiling temperature solvents. A variety of MAPbBr_3_ and MAPbI_3_ micropatterns (parallel lines, hexagons, and circular arrays) with controlled crystallinity and intrinsic photoelectric properties were fabricated. Moreover, micropatterns produced by the solvent-assisted gel printing method kept almost the same absorption and PL properties as usual HP thin films.^104^

Another HP 3D periodic structures are photonic crystals, which are interesting examples of structures with sophisticated morphologies and unique properties. By definition, a photonic crystal is a hierarchically porous ordered structure with areas of high and low refractive indices. Such structures hold great potential in electronic, optical and photocatalytic utilizations due to the slow-light effect which enhances the light absorption of the material.^105,106^ The fabrication of HP-based photonic crystals was first reported by the Tüysüz group. MAPbX_3_ (X = Cl, Br) based photonic crystal thin films with controllable porosity and thicknesses between 2 and 6 μm were successfully prepared. The group used the colloidal template method which entails assembling polystyrene opal templates, infiltrating methylammonium liquid precursor, and removing polystyrene spheres using toluene. Varying the size of the PS spheres, the position of the photonic stop band can be easily tuned through the visible spectrum as well as band gap energies by adjusting halide ratio.^107^

### 4.4 Charge Separation

Upon interaction of HPs with light, photoexcited charge carriers are generated. For efficient exciton formation, the energy of the incoming irradiation should be greater than or equal to the band gap energy of the HP. Photoexcited e^–^ and h^+^ can further migrate to the surface and drive electrochemical and catalytic reactions producing useful chemicals. A large portion of the photoexcited carriers undergoes recombination either on the surface of the catalyst or in the bulk through radiative (photo) and/or nonradiative (thermal) processes. HPs are considered a superb photocatalyst due to their good optical and transport properties such as large carrier diffusion lengths, long lifetimes, low trap densities, high charge carrier mobility, and low recombination rate.^108,109^ The path length that photoexcited e^–^ and h^+^ travel from generation to recombination is considered the carrier diffusion length. A long electron–hole diffusion length usually requires several conditions such as a long carrier lifetime, high carrier mobility, and low recombination rates, which all depend on material properties such as the exciton binding energy.

Zhang and co-workers observed extra-long electron–hole diffusion lengths in MAPbI_3-x_Cl_*x*_ of 380 μm under 1 sun illumination in crystals with varying amounts of chlorine. The group further deduced that there were two prevailing factors for electron–hole recombination and transfer: first, an increase in the density of trap-states, and, second, a reduction of the VB level by incorporation of chlorine ions.^110^ Improvement of the carrier diffusion length was also demonstrated by Zhumekenov et al. They reported a remarkable enhancement in electron–hole transport in FAPbX_3_ single crystals. FAPbBr_3_ displayed a 5-fold longer carrier lifetime and 10-fold lower dark carrier concentration than those of MAPbBr_3_ single crystals. The carrier diffusion lengths were found to be longer than 6.6 μm for FAPbI_3_ and 19.0 μm for FAPbBr_3_ single crystals.^111^

**Figure 13:**
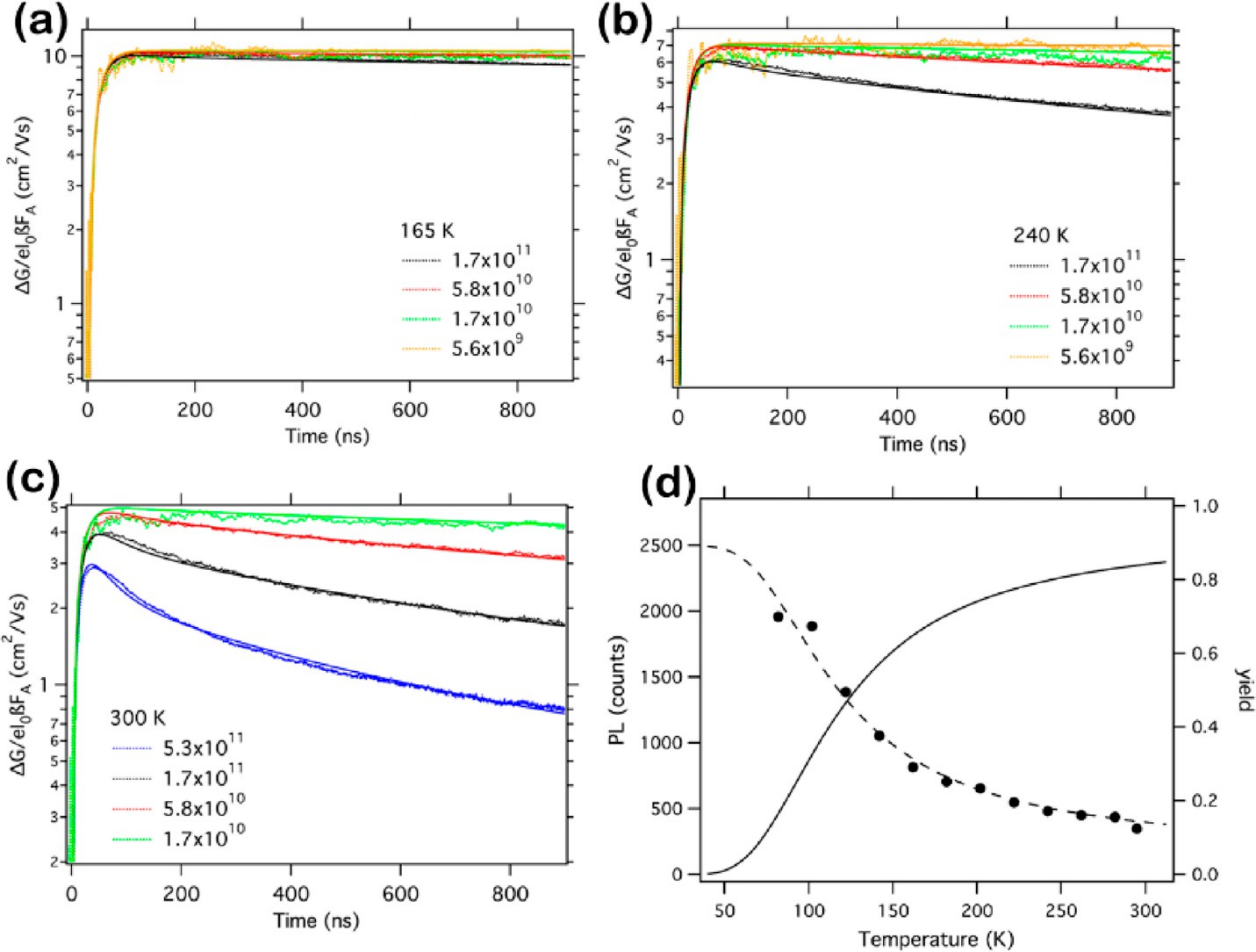
Normalized intensity photoconductance traces vs. time. Charge carrier dynamics obtained using various excitation intensities at (a) 165 K, (b) 240 K, and (c) 300 K, and (d) PL of MAPbI3 on Al2O3 vs temperature (circles) detected by integrating over the emission band on optical excitation at 514 nm. The dashed line represents an exponential fit to the data points yielding binding energy of 32 ± 5 meV. The solid line shows the yield of charges on assuming that thermal ionization is the only nonradiative decay channel. Reproduced from ref (113). Copyright 2014, American Chemical Society.

Yang et al. studied the surface recombination in MAPbBr_3_ single crystals using broadband transient reflectance spectroscopy. They found that electron–hole dynamics depend on the surface recombination and carrier diffusion from the surface into the bulk. Moreover, the surface recombination velocity was estimated to be 3.4 ± 0.1 × 10^–3^ cm s^–1^. The obtained value was 2–3 orders of magnitude lower than that of many unpassivated semiconductors used in solar cell applications.^112^ Savenije and co-workers investigated the temperature dependence of the carrier generation, mobility, and recombination in MAPbI_3_ by microwave photoconductance and PL techniques. The temperature-dependent yield of highly mobile electron–hole pairs was 6.2 cm^2^ V^–1^ s^–1^ at about 32 meV and was obtained maintaining the temperature at 300 K in the tetragonal crystal phase. Moreover, reducing the temperature to 160 K led to a reduction in phonon scattering by Σμ = 16 cm^2^ V^–1^ s^–1^ (Σμ is the sum of the electron and hole mobility) and an increase in charge carrier mobilities following a T^–1.6^ dependence. It is interesting to note that while Σμ was increasing, γ was decreasing by a factor of 6. This observation led to the conclusion that the electron–hole recombination in MAPbI_3_ was temperature-activated (Figure 13).^113^

Kanemitsu et al. investigated intensity-dependent photocarrier recombination and relaxation processes in MAPbI_3_ thin films using time-resolved PL and transient absorption methods. The PL intensity right after excitation displayed double-fold dependence on the excitation intensity implying that radiative recombination of photoexcited carriers is the primary process for PL and the exciton model was not as accurate at room temperature.^114^

The charge separation properties of all-inorganic HPs have also been heavily explored. De Jong et al. examined ultrafast carrier dynamics in CsPbBr_3_ NCs via pump–probe transient-induced absorption spectroscopy. CsPbBr_3_ NCs (8.6 nm) displayed the formation of multiple exciton complexes, with a multiplicity higher than two. The Auger time constants were defined for biexciton (τ_2_ ≈ 85 ps) and higher-order exciton complexes (τ_*x*>2_ ≈ 35–70 ps). Based on that, it was determined that CsPbBr_3_ NCs have a higher than two degeneracy of the ground state which is adverse for light-emitting diode and photovoltaic cell applications due to unsuppressed Auger recombination but favorable for possible laser applications.^115^

In addition to single crystal organic and all-inorganic HPs, carrier dynamics in their derivatives have been also studied. Especially for the photocatalytic application of HPs, charge separation is one of the key points that should be taken into consideration. There are many examples of HP-based nanocomposites, where it has been shown that the introduction of a cocatalyst (QDs, metal nanoparticles, MOFs) improves the separation of carriers.^116−118^ As an example, Rathore et al. demonstrated the successful synthesis of a quasi-type II heterojunction between N-doped carbon dots and CsPbBr_3_ NCs. They found that N doping of carbon dots introduces trap states below the conduction band which improves band alignment and charge transfer in the heterojunction.^119^ Wang et al. synthesized a Z-scheme 0*D*/2D CsPbBr_3_ QDs/Bi_2_WO_6_ nanosheet composite. Appropriate band alignment promoted charge separation and suppressed radiative recombination in CsPbBr_3_ QDs which was proven by PL studies. Superior CO_2_ photocatalytic reduction performance was observed compared with pristine CsPbBr_3_ QDs and Bi_2_WO_6_ nanosheets, maximum production of CO and CH_4_ over CsPbBr_3_ QDs/Bi_2_WO_6_ was 50.3 μmol g^–1^ h^–1^ which is 9.5 times higher than individual materials.^120^ Similarly, a heterostructure between CsPbBr_3_ nanocrystals and Bi_2_O_2_Se nanosheets was obtained. Increasing amount of Bi_2_O_2_Se was related to an increase in photoluminescence quenching, demonstrating charge transfer from the conduction band of CsPbBr_3_ to that of Bi_2_O_2_Se.^121^ Another example of successful charge separation was demonstrated by Cheng and co-workers. They boosted the photocatalytic CO_2_ reduction on CsPbBr_3_ NCs with Ni metal complexes. The PL analysis confirmed that the preparation of CsPbBr_3_ NCs/Ni(tpy) nanocomposite effectively suppressed charge recombination and promoted charge separation. The photocatalytic activity was further improved achieving 431 μmol g^–1^ h^–1^ of CO and CH_4_ which is a 26-fold increase compared to pristine CsPbBr_3_ NCs.^64^

### 4.5 Adsorption and Desorption

Adsorption and desorption processes on the surface of HPs depend on the crystallographic plane.^122^ The (001) plane of HPs has proven to be very stable (against air and moisture) and for that reason it has been extensively modeled. The (001) plane is terminated by either a PbX_2_ surface or an AX surface (X = halogen). Because these surfaces do not have midgap or shallow surface states, they contribute to long charge carrier lifetimes.^123^ The coordination numbers of surface atoms along with the number of dangling bonds determine the stability of different stoichiometric surface configurations. Due to the lack of experimental data, adsorption and desorption processes on the surface of HPs have been mainly obtained by computational simulations. Little information about surface interactions with adsorbate molecules has been collected for HPs. Due to various surface defects including dangling bonds and vacancies, HP surfaces have abundant adsorption and desorption sites. These HP surface sites participate in the interaction with H_2_O, O_2_, and other gaseous molecules.

One of the major bottlenecks of HPs for a wider application is their moisture sensitivity. HPs easily adsorb water and subsequently degrade, losing their unique properties. Understanding the degradation process of HP surfaces can help in finding solutions for their retention and application in numerous fields. Koocher and co-workers studied the interaction between (001) surfaces of MAPbI_3_ and water by DFT. They discovered that the orientation of the methylammonium cations close to the surface strongly influences water adsorption properties. Water molecules can enter hollow sites on the surface and get trapped, depending on the methylammonium orientation. The authors further showed that moisture stability can be improved by adjusting dipole orientation by poling or interfacial engineering.^124^ Tong et al. went further and determined the water adsorption energy on the MAPbI_3_ (001) surface which resulted to be about 0.30 eV. They proved Koocher’s observation, claiming that due to the huge interspatial distance in the MAPbI_3_ structure, water molecules can easily infiltrate into the surface, leading to full decomposition of the material. Moreover, the distortion induced by water strongly influenced the electronic structure and hence optical and electronic properties.^125^ Ma and co-workers proved that the adsorption and desorption are key parameters in maintaining water stability of PEA_2_PbI_4_ NCs. They demonstrated that PEA_2_PbI_4_ degrades following a desorption process, where PEA^+^ desorbs from the perovskite. However, in high concentrated (>0.15 M) PEA^+^ water solution, PEA_2_PbI_4_ stabilizes upon the adsorption process of PEA^+^ on the perovskite. Particularly important were the findings that the desorption and adsorption processes were recyclable.^126^

**Figure 14:**
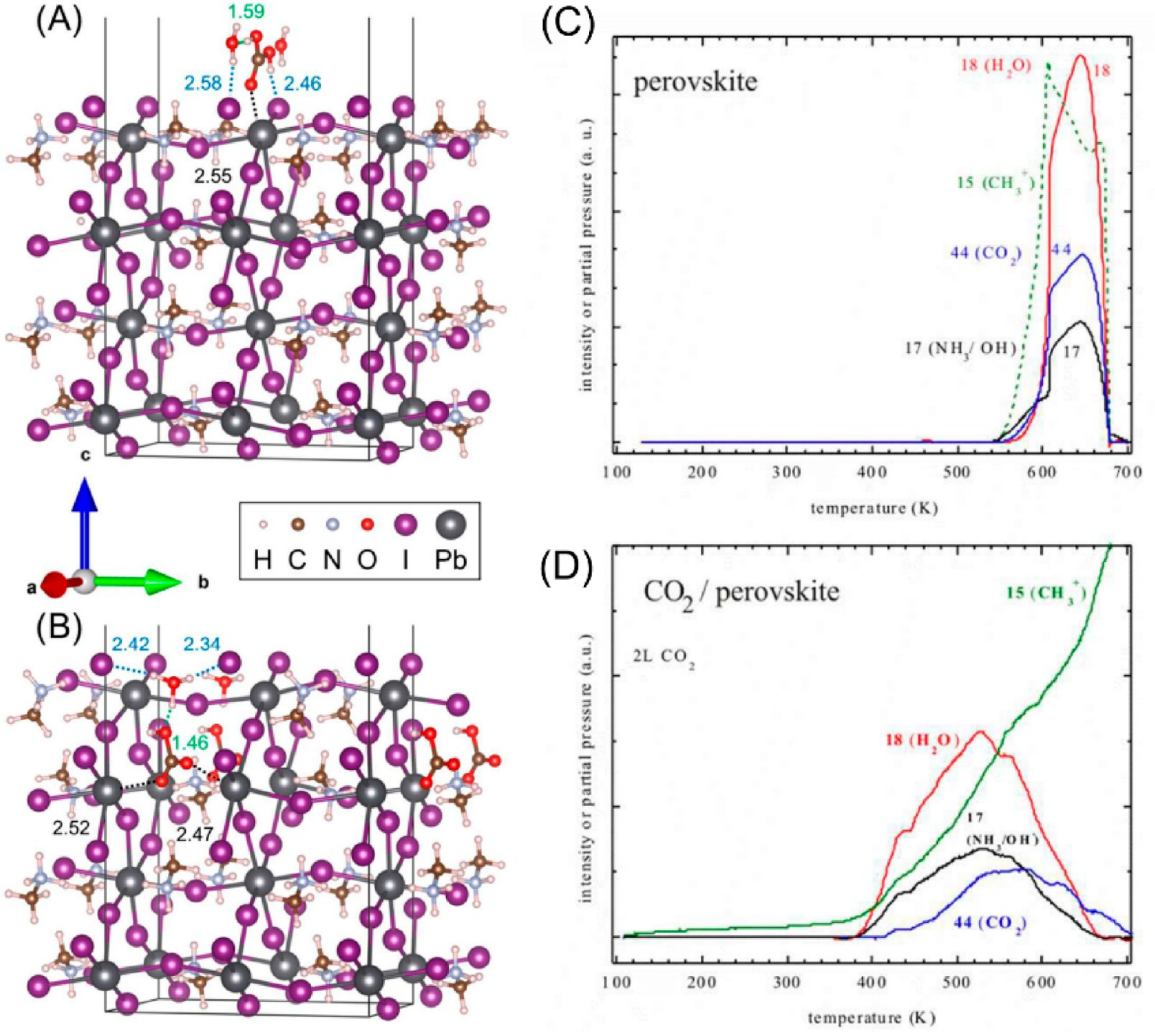
HPs model (a) H_2_O–H_2_CO_3_ complex and (b) defect structure with embedded H_3_O^+^ and HCO_3_^–^. The box represents the simulation cell which contains an H_2_O–H_2_CO_3_ complex or H_3_O^+^ – HCO_3_^–^ ion pair. Due to the visualization, additional species can be observed. (c) Multimass thermal desorption spectroscopy of perovskite thin film and (d) after a 2L dose of CO_2_ on a 2nd sample. Reproduced with permission from ref (127). Copyright 2018, The Royal Society of Chemistry.

Since the application of HPs was extended to the CO_2_ photocatalytic field, understanding the adsorption and desorption of CO_2_ and H_2_O is of crucial importance. The formation of adsorbed CO_2_ species on HP surfaces can be considered a rate-limiting and selectivity-determining step during photocatalytic reduction. It happens because of the high reorganizational energy between bent anion radical CO_2_^•–^ and linear CO_2_ molecules. Nayakasinghe and co-workers experimentally and theoretically characterized unusual adsorption kinetics of CO_2_ on MAPbI_3_ using ultrahigh vacuum and DFT calculations (Figure 14). They experimentally showed CO_2_ desorption at temperatures of 640 K on MAPbI_3_ thin films. DFT calculations suggested formation of a defect structure, where CH_3_NH_3_^+^ was replaced by H_3_O ^+^ and I^–^ by HCO_3_^–^. Furthermore, DFT predicted the formation of carbonic acid (H_2_CO_3_) or bicarbonate (HCO_3_^–^) from adsorbed CO_2_ and H_2_O which were trapped inside a defected perovskite structure.^127^ Xu et al. investigated CO_2_ photocatalytic reduction on CsPbBr_3_ and Co, Fe-doped CsPbBr_3_. The CO_2_ adsorption/desorption kinetics and CO_2_ reduction to CH_4_ mechanisms were examined. The obtained data suggested that the Co and Fe doped CsPbBr_3_ have better adsorption of the intermediate adsorbate species, promoting the activation of CO_2_* and enhancing photocatalytic activity. The better photocatalytic performance for Co and Fe doped CsPbBr_3_ was further confirmed by charge and density of states analysis.^128^

### 4.6 Selectivity

As discussed in previous sections, the interaction of HPs with light irradiation leads to the formation of photoexcited electron–hole pairs. These photoexcited carriers can further selectively participate in various photocatalytic reactions. HPs have been known for selective reductions to CH_4_ from H_2_O and CO_2_ and selective oxidations to formic acid from methanol. The selectivity of HPs toward target products usually arises upon modification of band gap energies, size, morphology, chemical composition, solvent effects, and introduction of suitable cocatalysts. Usually, the main products of photocatalytic CO_2_ reduction on HPs are CO and CH_4_, achieved upon efficient utilization of photoinduced holes on different oxidations of e.g. water or scavengers.

Pioneering work on colloidal CsPbBr_3_ QDs for selective CO_2_ reduction was published by Prof. Hou and colleagues.^23^ As-prepared CsPbBr_3_ QDs (3–12 nm) demonstrated quantum size effects resulting in tunable band gap energies and PL emissions covering the visible region. The CO_2_ photocatalytic reduction was performed in an ethyl acetate aqueous system using a 300 W Xe lamp with a standard AM 1.5 G filter for 8 h. The main products of the photocatalytic reaction were CO (4.3 μmol g^–1^ h^–1^), CH_4_ (1.5 μmol g^–1^ h^–1^), and H_2_ (0.1 μmol g^–1^ h^–1^). The CsPbBr_3_ QDs exhibited an average electron yield of 20.9 μmol g^–1^ for solar CO_2_ reduction with a high selectivity of over 99%. Xu et al., for the first time, used GO to prepare nanocomposites with CsPbBr_3_ for boosting CO_2_ photoreduction. Pristine CsPbBr_3_ QDs demonstrated CO_2_ reduction at a rate of 23.7 μmol g^–1^ h^–1^ with a high selectivity over 99.3%, producing CO (49.5 μmol g^–1^) and CH_4_ (22.9 μmol g^–1^) as main products and a small production of H_2_. However, upon the formation of the CsPbBr_3_ QDs/GO nanocomposites, the rate of electron consumption increased by 29.8%. Assigned to a better electron transport from CsPbBr_3_ to GO, the production of solar fuels was improved to 58.7 and 29.6 μmol g^–1^ for CO and CH_4_, respectively. Moreover, the stability was gradually improved up to 12 h under illumination conditions upon the introduction of GO.^24^

**Figure 15:**
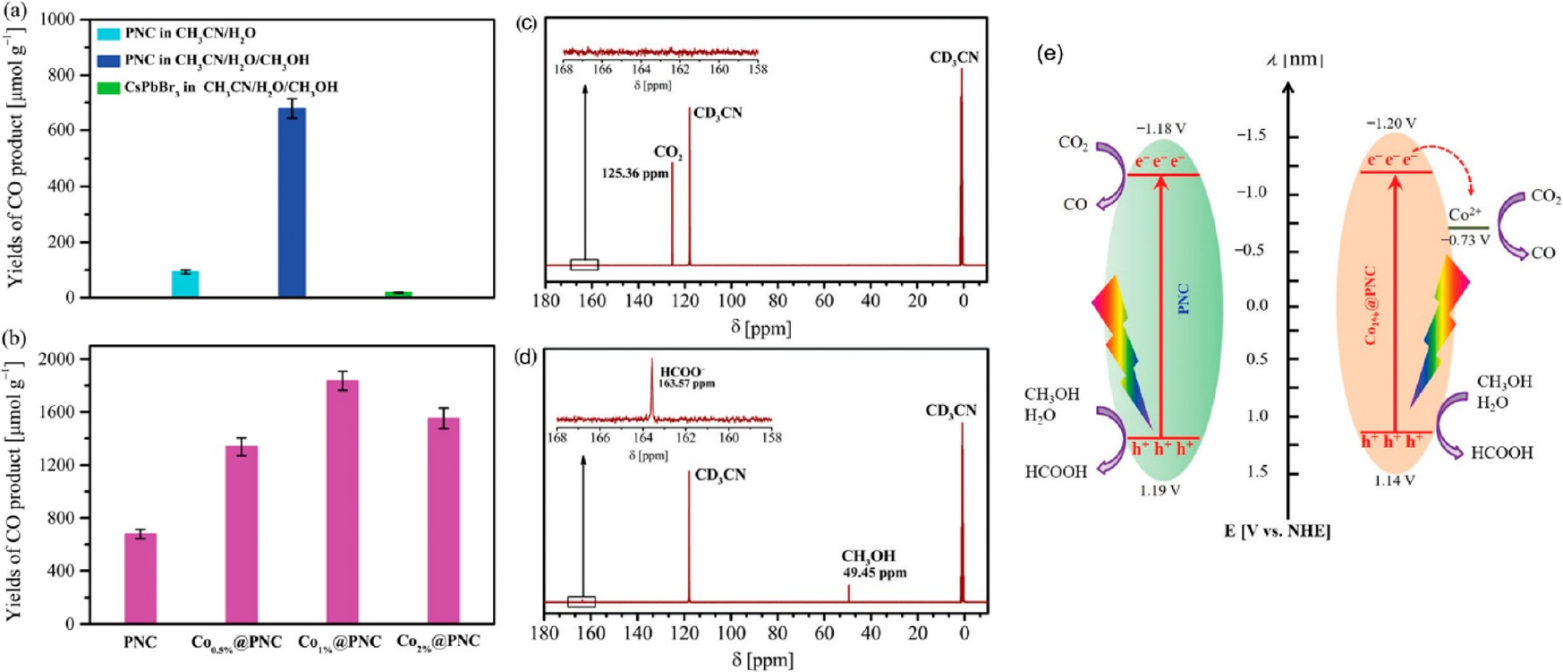
Results of photocatalytic CO_2_ reduction to CO (a) using CsPbBr_3_/Cs_4_PbBr_6_, and CsPbBr_3_ without and with CH_3_OH, (b) using CsPbBr_3_/Cs_4_PbBr_6_ and Co-doped CsPbBr_3_/Cs_4_PbBr_6_ with CH_3_OH (c) ^13^C NMR spectra for the liquid products ^13^CO_2_ and CH_3_OH and (b) CO_2_ and ^13^CH_3_OH as feedstocks and (e) schematic band structures diagram for CsPbBr_3_/Cs_4_PbBr_6_ and Co-doped CsPbBr_3_/Cs_4_PbBr_6_. Reproduced with permission from ref (130). Copyright 2020, The Royal Society of Chemistry.

Wan and co-workers explored CsPbBr_3_ QDs coupled with UiO-66(NH_2_) MOF. The as-synthesized nanocomposite showed an enhanced photocatalytic reduction of CO_2_ compared to pristine CsPbBr_3_ QDs and UiO-66(NH_2_). The CsPbBr_3_ QDs/UiO-66(NH_2_) nanocomposite selectively reduced CO_2_ to CO and CH_4_ in an ethyl acetate/H_2_O system, reaching an electron consumption rate of 18.5 μmol g^–1^ h^–1^. The performance of the nanocomposite was attributed to the improved electron transfer between UiO-66(NH_2_) and CsPbBr_3_ QDs, their large specific surface area, enhanced visible light absorption capacity, and improved stability.^129^ Dong et al. demonstrated successful selective photocatalytic reduction of CO_2_ to CO and CH_3_OH oxidation on CsPbBr_3_/Cs_4_PbBr_6_ nanocomposites. A photocatalytic experiment was carried out in an acetonitrile/H_2_O aqueous system. The CsPbBr_3_/Cs_4_PbBr_6_ nanocomposites alone achieved a CO yield of 93 μmol g^–1^. Under the same irradiation conditions, the addition of a small amount of CH_3_OH to the reaction system brought a significantly enhanced yield of CO to 678 μmol g^–1^ due to accelerated hole consumption. Furthermore, CH_3_OH was oxidized into formic acid which was confirmed by ^13^C NMR spectroscopy (Figure 15).^130^

Trying to move away from toxic CsPbBr_3_ materials, Kuang and co-workers^71^ synthesized Pb-free double HPs for photocatalytic CO_2_ reduction. Cs_2_AgBiBr_6_ NCs were prepared by the conventional hot injection method, exhibiting certain stability against moisture, light, and temperature. Their Cs_2_AgBiBr_6_ NCs selectively photocatalytic reduced CO_2_ to CO (5.5 μmol g^–1^) and CH_4_ (0.65 μmol g^–1^), overall representing an electron consumption rate of 105 μmol g^–1^ during 6 h.

## 5 Important Considerations and Challenges

### 5.1 Reaction Conditions

The process of photocatalytic reduction of CO_2_ into clean fuels entails an extensive optimization of numerous interconnected process variables. Although the type and quality of the perovskite crystals in study play a crucial role in dictating the final products and the rate of their production, the reaction setup and operating conditions also have a significant impact on the process. These conditions include, but are not limited to, reaction temperature, pressure inside the reactor, type of incident light, use of hole scavengers, and catalyst concentration. This section will briefly highlight the different reaction conditions and their influence.

#### 5.1.1 Reaction Temperature

The overall reaction temperature is considered a main condition when performing photocatalytic reduction experiments due to its effects on the CO_2_ solubility, reactant adsorption on the surface of the photocatalyst, and products desorption, all influencing the reaction rate constant. For liquid phase reactions at a constant pressure, an increase in the reaction temperature leads to a decrease in the solubility of CO_2_ in water but an increase in most organic solvents such as methanol, ethanol, and propylene glycol.^131−133^ For this reason, a change in temperature can affect the levels of CO_2_ dissolved in the liquid reaction mixture and, in turn, change the quantity of CO_2_ available on the catalyst surface. Similarly, the use of higher temperatures at a constant pressure for gas-phase photocatalytic reactions means higher kinetic energy of CO_2_ molecules in the reactor volume but a decrease in their molecular density. The increase in kinetic energy increases the probability of CO_2_ interactions with the photocatalyst surface due to the increase in the collision frequency of the reactants. However, excessive energy can also have a negative impact of the adsorption of the reactants on the surface of the photocatalyst. On the other hand, lower temperatures in gas-phase reactions can slow down the rate of product desorption from the surface of the catalyst. This contributes to catalyst poisoning where the availability of active sites is diminished, and no new reactants can adsorb on the surface.^131^

#### 5.1.2 Reactor Pressure

**Figure 16:**
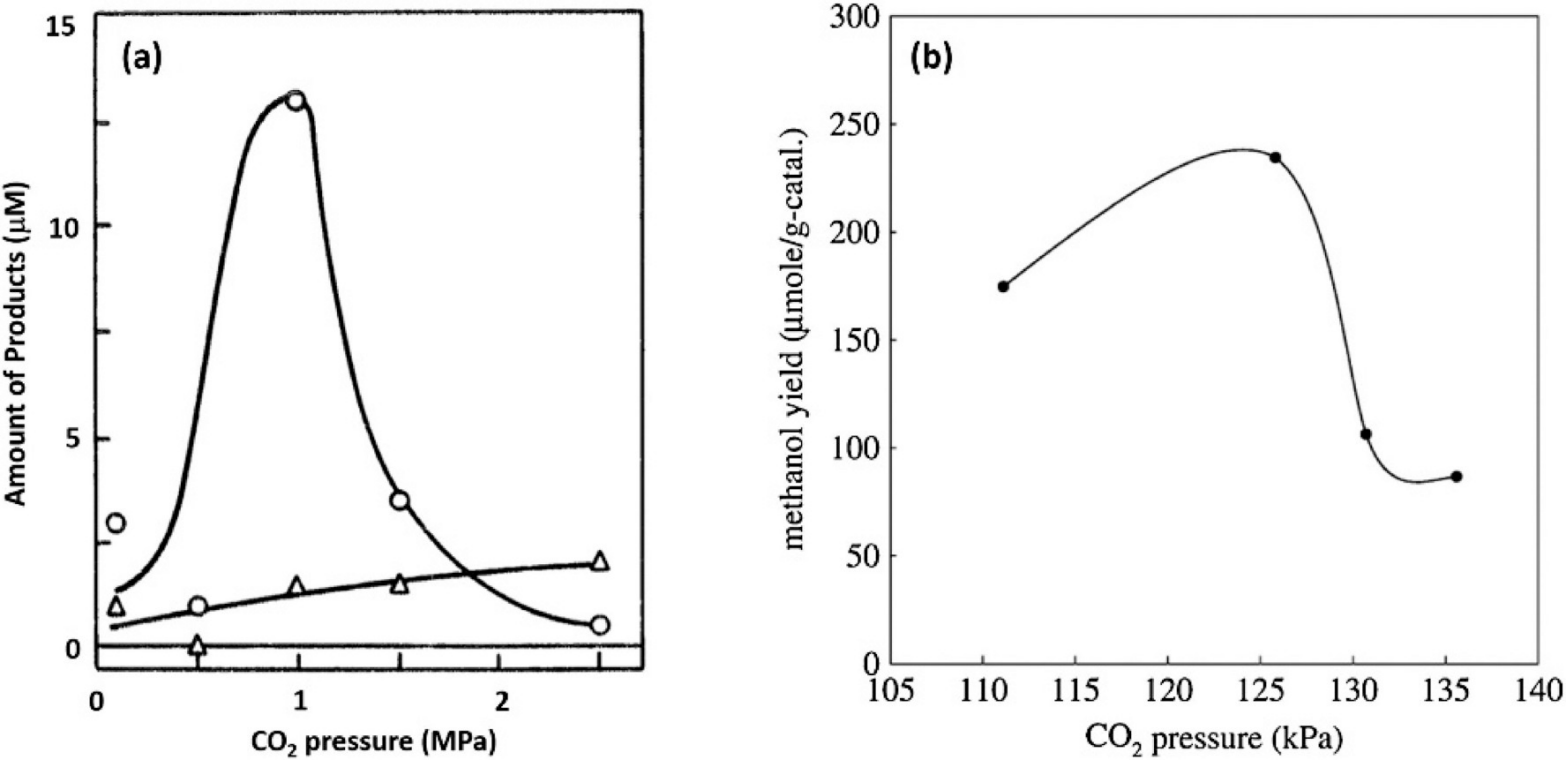
(a) Variation in HCOOH (Δ) and CH_3_OH (o) production with pressure for TiO_2_ in suspension. Reproduced with permission from ref (134). Copyright 1996, Elsevier. (b) CH_3_OH yield with pressure for Cu/TiO_2_ in suspension. Reproduced with permission from ref (136). Copyright 2002, Elsevier.

Although not heavily explored for gas-phase reaction with HPs, studies performed on other photocatalysts can show that the pressure of CO_2_ inside the photoreactor can have a significant impact on selectivity and production rates. For example, Mizuno et al. studied the effect of CO_2_ pressure on the activity of TiO_2_ for photocatalytic CO_2_ reduction in an aqueous solution of NaOH. They found that the rate of CH_3_OH production was directly proportional to CO_2_ pressure up to 1 MPa after which it decreased significantly.^134^ It was explained that the increase in CO_2_ pressure led to an increase in its solubility in the aqueous solution, thereby improving the adsorption of the main reactant on the surface of the catalyst. A similar observation was made by Koci et al. when the production of both, CH_3_OH and CH_4_, increased from 0.75 and 2.75 μmol g^–1^ to maxima of 1.5 and 4.5 μmol g^–1^, respectively, with a 20 kPa increase in pressure (Figure 16a).^135^ Tseng et al. further confirmed the existence of an optimal pressure after which the production of CH_3_OH decreases. The group found that the use of 125 kPa produced 230 μmol g^–1^ and a further increase of 10 kPa led to a decrease to 85 μmol g^–1^ (Figure 16b).^136^

#### 5.1.3 Wavelength, Intensity, and Duration of Incident Light

The type of light being used for photocatalysis has a significant effect on the efficiency of the process as well as the integrity of the photocatalyst itself. To begin, the photon energy of the incident light has to be greater than the band gap of the chosen photocatalyst to allow the electrons to be excited from the VB to the CB. Solar spectrum references are standardized by the American Society for Testing and Materials (ASTM) International organization. It has become customary that the lab-scale testing for solar cell and photocatalytic applications simulate the AM 1.5 G solar spectrum with an incident irradiance of approximately 1000 W m^–2^. This solar spectrum was derived based on the average annual solar irradiance at intermediate latitudes. The AM 1.5 D spectrum utilizes a 900 W m^–2^ irradiance from a direct beam from the sun in addition to a 2.5° circumsolar disk around the sun. Typically, referring to 1 sun of intensity for an AM 1.5 G solar simulator corresponds to 1000 W m^–2^.^137,138^

In addition to the choice of irradiance, one can filter out specific wavelengths of light depending on the desired conditions. Different suppliers provide a wide range of filters that can cutoff wavelengths below 320, 360, 380, 400, or 420 nm.^139^ The type of light being used, and its intensity play a very important role in the efficiency of the photocatalytic process. One main reason is the effect of the intensity on the stability of the HP. As an example, utilization of an AM 1.5G solar simulator seldom leads to the rapid breakdown of iodide-based HPs. It was described that iodide vacancies are generated upon photoexcitation allowing oxygen to be introduced into the perovskite structure.^140,141^ As a result, superoxides form and produce several decomposition products such as H_2_O, CH_4_, and PbI_2_ most commonly observed in organic lead iodide perovskites, in specific.^140,141^

The use of different light sources might cause the photocatalytic system to behave differently. For example, Bafaqeer et al. conducted a comparative study of a ZnV_2_O_6_/RGO/g-C_3_N_4_ heterojunction irradiated with a 200 W Hg lamp (150 mW cm^–2^) as a source of UV light and a 100 mW cm^–2^ solar simulator. The study reported that while in both cases the production of H_2_, CH_4_, and CH_3_OH followed a similar trend, the total rate of production was higher when solar light was used. The group attributed this improvement to a shift from a type I heterojunction behavior to a Z-scheme behavior under solar light.^142^

Another variable that can greatly affect the photocatalytic process is light intensity. Since the illumination intensity is directly proportional to the number of photons per unit area during a specific time interval, then it is logical to assume that the rate of product formation will also be proportional to intensity.^131^ Kumar et al. reported a significant increase in overall photocatalytic activity of CsPbBr_3_ composite when the light intensity was increased from 1 to 2 sun. This improvement in activity was also coupled with a shift in selectivity from CH_4_ to H_2_ and the group linked this observation to a change in reaction temperature due to heat generated from the light.^58^ While the intensity of the incident light can increase the rate of product formation, one main disadvantage is inducing the early decomposition of the perovskite.

Depending on the catalyst at hand, a high light intensity for a prolonged time duration can destabilize perovskite. Reaction time is another variable to be considered when planning out photocatalytic CO_2_ reduction experiments. Depending on the stability of the photocatalyst under the chosen conditions, the rate of gas production can either increase linearly with time or reach an optimum value after which the rate decreases and the curve plateaus about a maximum. Gao et al. studied the evolution of H_2_ and CO gases over the course of 90 min when a CoSn(OH)_6_ perovskite was illuminated with 300 mW cm^–2^ light source with a 400 nm cutoff filter.^143^ The study showed that the production rate of the two gases was higher at the start of the reaction and slowed down gradually to reach a plateau after approximately 90 min. The group correlated this decrease in rate to the instability of the photosensitizer used in the liquid-phase reaction ([Ru(bpy)_3_](PF_6_)_2_).

Although not deeply studied in literature, the theory of light soaking HPs has been slightly touched upon in solar cell research. The effects of light soaking on the performance of solar devices have often been correlated to changes in ion migration demonstrated by JV curves.^144,145^ Several groups have reported improvements in device performance upon preconditioning by light soaking for a short period of time before conducting the measurements. Bliss et al. noticed an increase in current after light soaking their devices for 20 min in addition to changes in the external quantum efficiency curve shape.^146^ Similarly, Pockett et al. noticed a clear improvement in current density in the JV curves after light soaking the device for only 3 min.^147^ Even though in some cases light soaking is considered beneficial for solar cell devices, it is also known to contribute to shortening the device lifetime. In short, it is hypothesized that the power conversion efficiency of solar cells is not constant but rather decreases with increasing illumination time mainly due to nonradiative recombination.^148^ Ebadi et al. highlighted the effect of light soaking in perovskite solar cells and hypothesized that this phenomenon is one of the various reasons for the instability of this material. The group confirmed that correlations for luminescence and open-circuit voltage measurements for an unstable system do not remain valid upon light soaking and related this to the formation of different phases with different contributions to the photocurrent.^149^ A similar observation was reported by Thi-Hai-Yen Vu et al. in their study about cesium lead halide perovskite solar cells.^150^

#### 5.1.4 Catalyst Loading Method

For gas-phase photocatalytic reactions, the loading method of the catalyst on support is of vital importance because of the role it plays in the distribution of light on its surface. Some researchers have opted to use monolith and fiber optic reactor designs to maximize the dispersion of catalyst and the surface illumination.^151^ While the reactor geometry is an important factor in this regard, this section will discuss the loading method in a cylindrical top-illuminated reactor and the effect of reactor geometry will be further discussed in Section 5.2.

To maximize the activation of the catalyst, a homogeneous distribution of the powder should be achieved where the largest possible area is subjected to light as well as reactants. Conventionally, the powder is placed on an inert support such as glass and flattened out to increase dispersion.^152^ Alternatively, to ensure better dispersion and prevent particle agglomeration, a quartz filter paper can be used to evenly spread dry catalyst powder and remove any excess. It is also possible to disperse the catalyst in a volatile solvent, cast it on the filter and dry the solvent to provide a homogeneously distributed layer of material.^153^

While methods of homogeneously dispersing perovskite powders on substrates vary, some of the common ways reported in literature of doing so include spin coating and drop-casting.^18,154,155^ Zuo et al. illustrated a common method for in situ synthesis of perovskite films on a substrate by drop-casting a solution of MAPbI_3_.^156^ While common in photoelectrocatalytic (PEC) applications and solar cell fabrication, little research has been gathered on the efficacy of such methodologies in photocatalysis. Chen and co-workers were able to achieve a good dispersion of CsPbBr_3_ perovskite heterostructures by coating them on a quartz substrate by the drop-casting method.^157^ Alternatively, some researchers have opted to chemically improve the dispersion of perovskite crystals by coating them on cocatalytic supports such as graphene, silica, or zeolites. This approach has shown an improvement in charge separation and an increase in the number of surface active sites and is discussed in more detail in literature.^158,159^

#### 5.1.5 Addition of Hole Scavengers

The most abundant electron donor for photocatalytic reactions is H_2_O. The means of addition of H_2_O to a gas-phase reactor varies in the literature. Saturating CO_2_ with H_2_O by passing the gas through a bubbler is an easy and common way often reported.^160−163^ Alternatively, some consider adding a specific amount of H_2_O volume into the photoreactor and allowing it to saturate slowly while purging with CO_2_. A more controlled way of H_2_O addition is injecting liquid at a specific rate, for example using a microsyringe pump in parallel with CO_2_ purging. This can give a better estimation of the humidity inside the reactor as well as ensure vapor saturation with CO_2_.^21^ The different methodologies of incorporating H_2_O into the system could be expanded to include different hole scavengers as well.

The effects of adding different hole scavengers such as triethanolamine and triethylamine have been heavily explored in literature.^164^ Wang et al. performed photocatalytic CO_2_ reduction experiments on a heterojunction between Cs_2_SnI_6_ perovskite nanocrystals and SnS_2_ nanosheets in the gas-phase. The reactor used for the experiments was filled with CO_2_ and injected with 5 μL of H_2_O and 5 μL of CH_3_OH. To further study the effect of triethanolamine as a hole scavenger, the group collected transient absorption spectra and observed that the decay was retarded and the electron lifetime prolonged.^165^ Since most of the scavengers used are liquid themselves, it has become more common to use hole scavengers when conducting liquid-phase photocatalytic experiments. The choice of catalyst and solvent depends on the stability of the perovskite in those solvents. For example, Gao et al. performed liquid-phase photocatalytic CO_2_ reduction of CoSn(OH)_6_ nanocubes in the presence of a 6 mL mixture of acetonitrile, H_2_O, and triethanolamine (4:1:1 ratio). High-purity CO_2_ was purged through a solution containing 0.1 mg of the photocatalyst prior to illumination and the resulting reaction produced approximately 19.3 mol of CO after 90 min.^143^

#### 5.1.6 Effect of Adsorbed Organic Species

As discussed in Section 3.2, different methodologies for the synthesis of HPs often require the use of different types of organic solvents such as dimethylformamide or acetonitrile. In addition, hot injection or LARP method requires the use of different organic capping ligands such as oleamide, oleylamine, or oleic acid to produce high-quality and stable QDs. The subsequent washing steps do not always succeed in removing all the solvents and excess of capping ligands which, in turn, may result in further adsorption of volatile organic compounds on the surface of the catalyst.^86^

**Figure 17:**
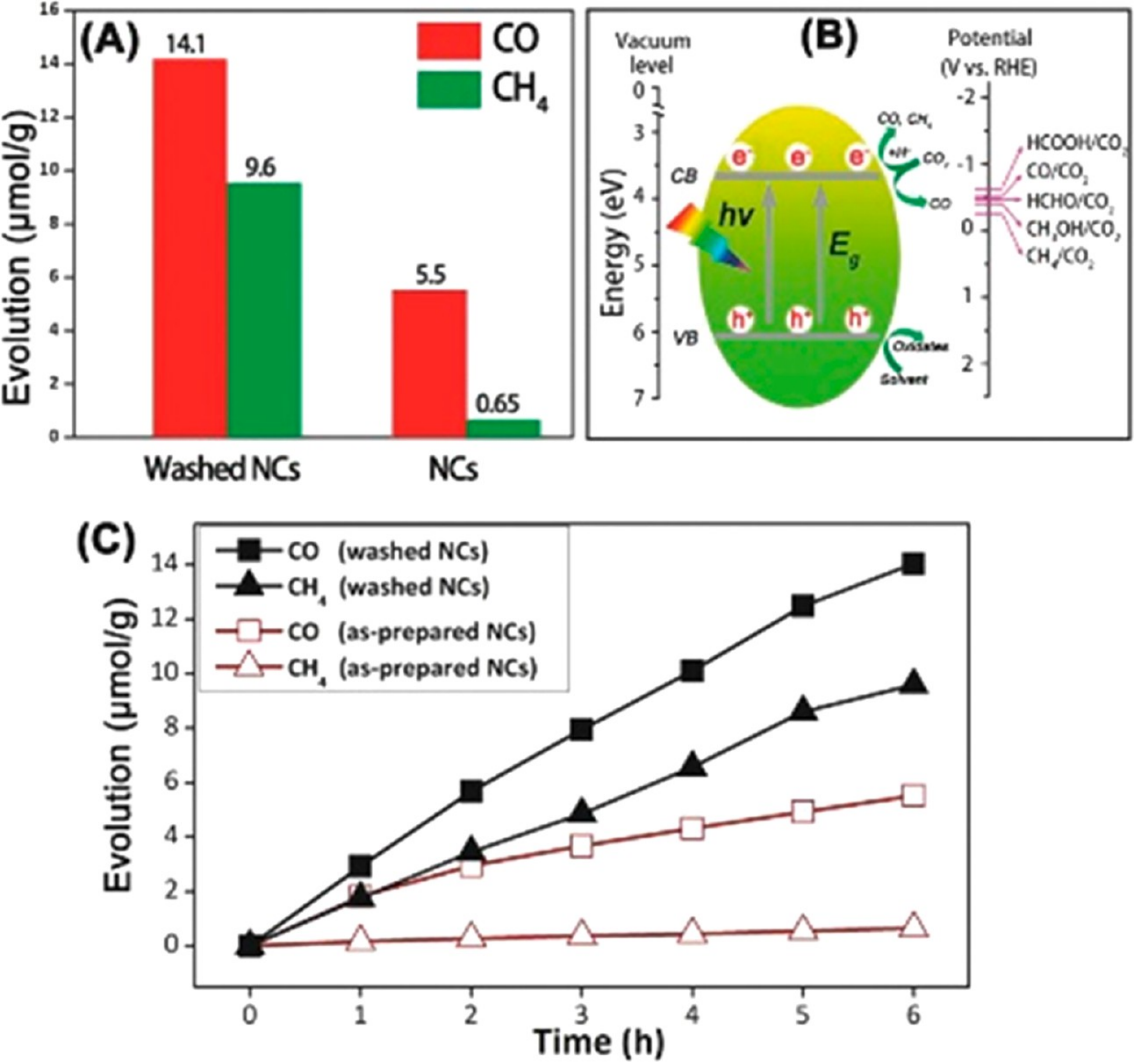
(a) Evolution of CO and CH_4_ from as-prepared and washed NCs. (b) Schematic representation of the possible photoreduction reactions on the surface of Cs_2_AgBiBr_6_ NCs. (c) Evolution of CO and CH_4_ gases for as-prepared and washed Cs_2_AgBiBr_6_ NCs with respect to time. Reproduced with permission from ref (71). Copyright 2018, John Wiley and Sons.

The presence of surface organic species complicates the photocatalytic process due to several reasons. First, long-chain organic species can block surface active sites and inhibit the adsorption of some reactants on the catalyst; therefore, they hinder charge transfer between perovskites and reactants.^100^ Zhou et al. were able to design a washing procedure that removes the capping ligands while maintaining the structural integrity of Cs_2_AgBiBr_6_ nanocrystals. As shown in Figure 17, careful washing of the NCs led to an improvement in the production of both CO and CH_4_ after 6 h of irradiation (AM 1.5G filter, 150 mW cm^–2^). The authors explain that ligands act as a charge barrier between the catalyst and the reactants.^71^ Numerous studies have attempted to optimize the washing process to produce stable perovskites with the least amount of capping ligands.^166−169^

Organic residues, capping ligands, and adventitious carbon can undergo different redox or decomposition reactions into carbon-based products. For example, many organic solvents such as dimethylformamide (DMF), dimethyl sulfoxide (DMSO), and acetonitrile undergo photolysis during the photocatalytic reaction and produce H_2_, CH_4_, or CO. Therefore, the identification of the origin of the measured products in the photocatalytic reactions becomes more difficult and can lead to false positives that require careful consideration.^164^

### 5.2 Photoreactor Design

Although scarcely highlighted in literature, the design of a robust photocatalytic reactor with minimum interference on the process itself is indeed a challenge. Despite research being heavily focused on quantitatively comparing different photocatalysts to one another under identical photocatalytic reaction conditions, not much attention has been paid to the large impact that the choice of reactor materials and light source configuration on the photocatalytic process.

The design of the photoreactor itself heavily depends on the chosen experimental parameters discussed in Section 5.1. There are general guidelines and conditions to be met when designing a photocatalytic reactor to maximize the overall efficiency and eliminate possible false positive results of the process. For example, the optical window should ensure the transmittance of the chosen light spectrum, the material of construction should be robust, chemically resistant, inert, and allow proper heat exchange, and the overall geometric configuration should allow proper flow of gases while minimizing air leakage into and product leakage out of the reactor.

As discussed in Section 5.1.3, the type of incident light dictates the wavelength range and intensity of the photons being used during the photocatalytic process. However, the photoreactor configuration, as well as the optical window through which the light is designed to pass, can heavily influence the actual properties of the photons reaching the surface of the photocatalyst. The light can either be chosen to be internal or external to the photoreactor. In any case, the direction of the incident light dictates the surface of the reactor that must be able to allow the passage of light through. That surface is commonly made from borosilicate glass or quartz (or fused silica) glass. In case the experimental conditions require the use of UV light, then the latter is the optimal choice since it has good transparency in that range.^170^ The remaining surfaces of the photoreactor can also be made of the same type of glass or stainless steel. For example, Wang et al. used a cylindrical stainless-steel vessel with a quartz glass window to pass the light for their gas-phase CO_2_ reduction experiments.^171^ On the other hand, Adekoya et al. chose to have the whole photoreactor as a jacketed vessel made of quartz glass and to embed their light source in a quartz tube inside the photoreactor itself.^172^ Kumar et al. performed their photocatalytic reactions of CsPbBr_3_ composites in a gastight glass photoreactor with a quartz window at the top.^21^

**Figure 18:**
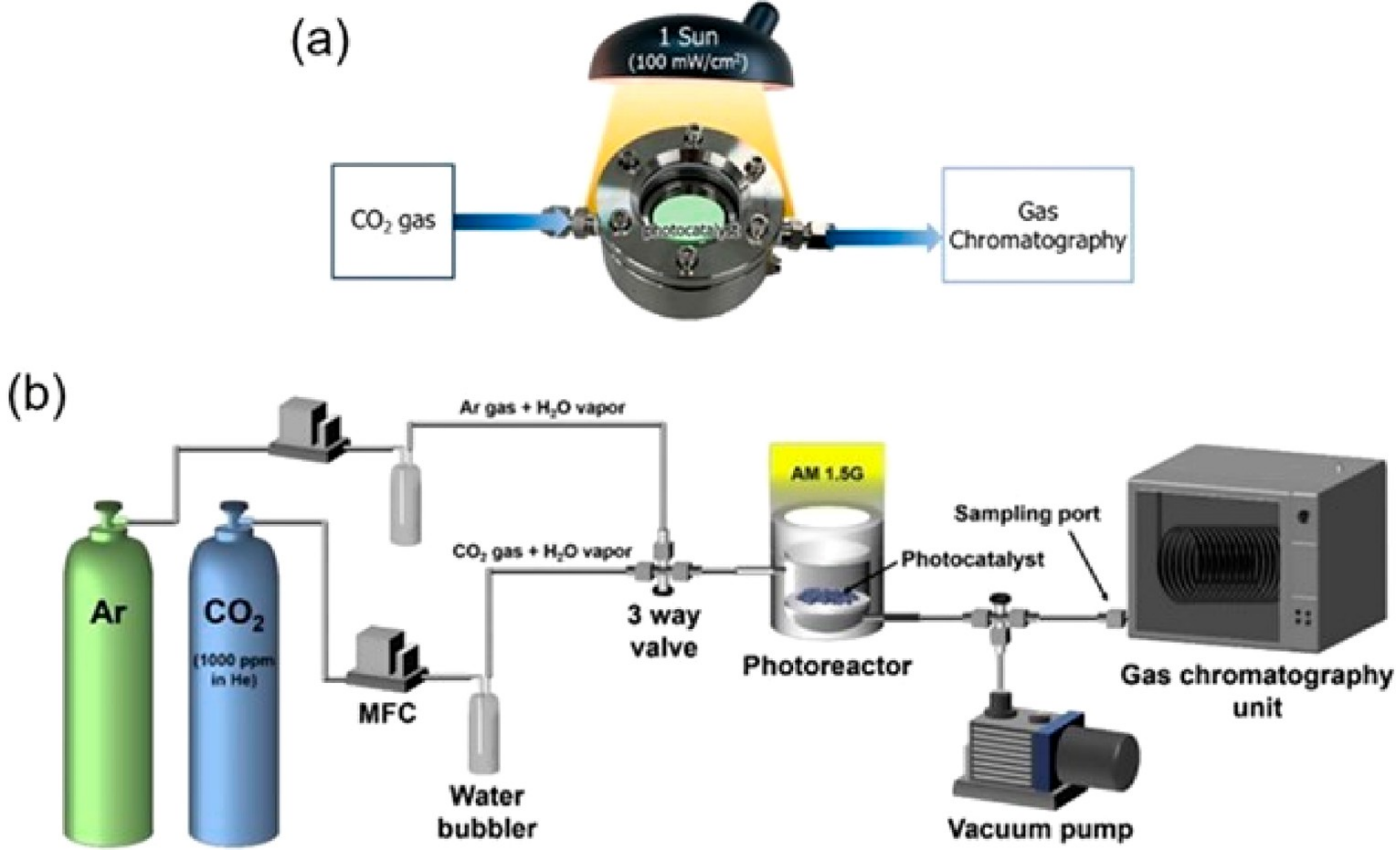
(a) Simplified schematic of the setup with a stainless-steel photocatalytic reactor. Reproduced with permission from ref (173). Copyright 2021, Multidisciplinary Digital Publishing Institute. (b) Detailed layout of a typical continuous flow photocatalytic CO_2_ reduction setup. Reproduced with permission from ref (160). Copyright 2017, Elsevier.

To study the efficiency of photocatalytic CO_2_ reduction using MAPbBr_3_ QDs, Lee et al. used a homemade stainless steel photoreactor with a quartz window. The catalyst was spread on a glass substrate and inserted into the chamber which was then purged with CO_2_ and sealed (Figure 18a).^173^ Sorcar et al. have clearly illustrated one of the common ways in which a photocatalytic CO_2_ reactor setup is typically designed (Figure 18b).^160^ The group used argon as an inert gas for purging before starting the reaction. CO_2_ gas was saturated with H_2_O by passing the gas from the cylinder through a water bubbler continuously into the reactor. The gas flow rate was maintained at 1 mL min^–1^ and was analyzed every 30 min by a gas chromatograph. In addition to that, air leakage into the reactor can minimize the production because excess O_2_ can take up some of the photogenerated electrons.

Even with a completed design for a photoreactor setup, changes and alterations to the existing lines and equipment are necessary at times. Any alterations might risk in line breakage (stainless steel, copper, or plastic tubing) and induce damage to valves in place and connections associated with mass flow controllers (MFC). In their work, Fresno et al. were able to easily set and accurately control the temperature, humidity, and flow rates in their photoreactor system using a Bronkhorst Controlled Evaporation and Mixing (CEM) unit and flow controllers. Depending on the inlet flow rate of the H_2_O and CO_2_ as well as the temperature of the CEM, the group was able to achieve a 7.25 CO_2_:H_2_O molar ratio which was purged for 1 h through the reactor to saturate their catalyst.^174^ Both, CO_2_ and Ar (as an inert) were passed through a gas-flow controller while distilled H_2_O from a tank was pressurized to pass through a liquid-flow controller. The gas and liquid mix in the CEM which is set to a specific temperature and then flowed to the reactor. The output of the reactor is analyzed by a gas chromatograph.^175^

**Figure 19:**
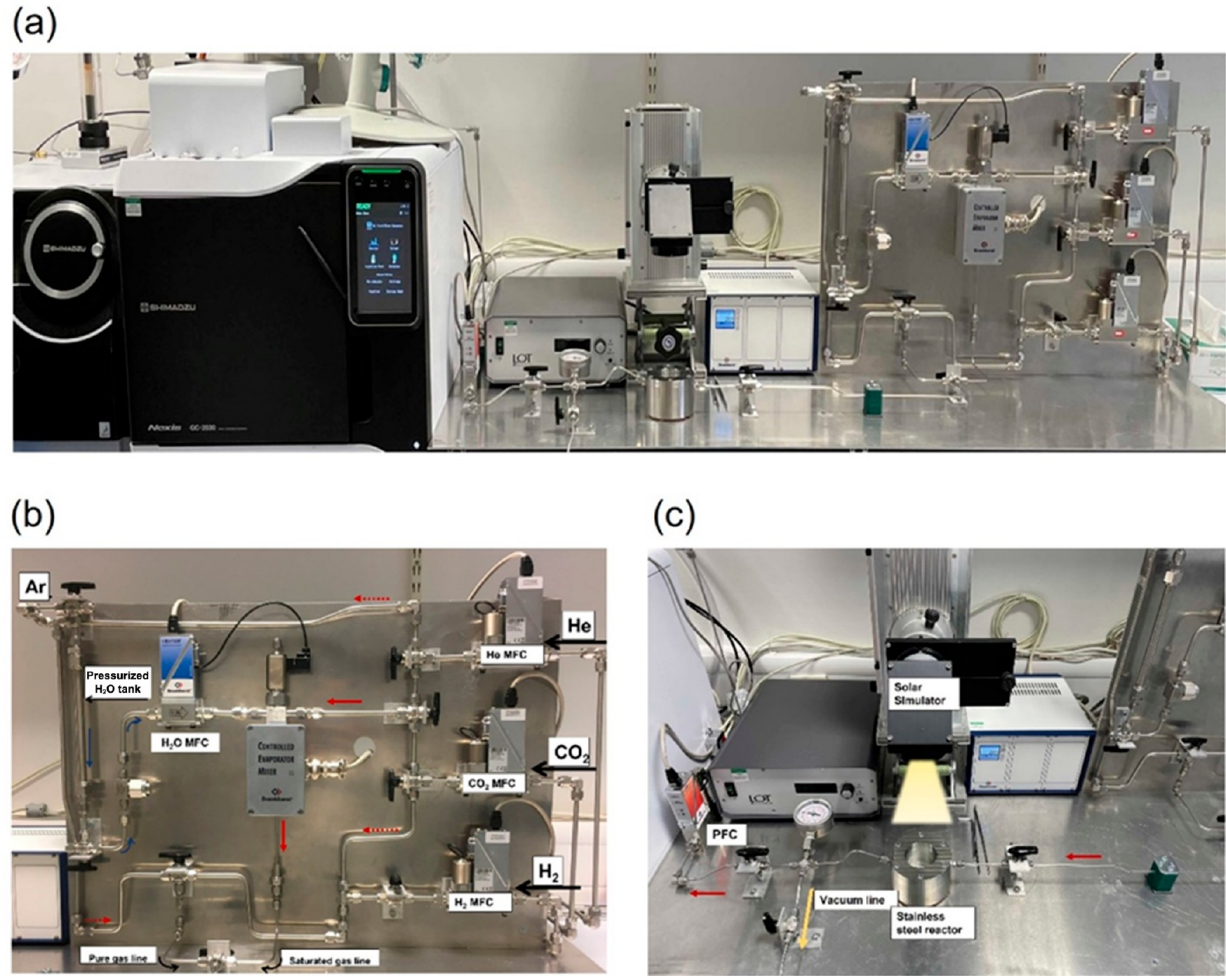
(a) Photoreactor setup for CO_2_ reduction and hydrogenation reactions from the Eslava group at Imperial College London. (b) Close-up of mass flow controller configuration before the reactor and (c) the photoreactor.

Building on the design from Fresno et al., our group has set up a controlled rig for photocatalytic CO_2_ reduction and hydrogenation reactions (Figure 19a). The overall setup contains 4 MFCs placed in parallel (Figure 19b) to control the flow of He inert gas for purging, CO_2_ for reduction reactions, H_2_ for hydrogenation reactions, and H_2_O(l) as an electron donor for reaction with CO_2_. For photocatalytic CO_2_ reduction experiments, the reactor can be purged with pure He gas for some time to remove the air inside. He inert gas is also used to perform control experiments (Figure 19c). A CEM evaporator is coupled to the MFC for H_2_O(l) and the gas flow to create a controlled humid atmosphere inside the reactor. A stainless-steel photoreactor with valves at the inlet and outlet is used in continuous or batch mode for both gas- and liquid-phase reactions. The top outlet of the reactor is connected to a vacuum pump for reactor evacuation as well as to a back-pressure controller to control the internal reactor pressure. The products are then analyzed by gas chromatography and/or mass spectroscopy.

As discussed in Section 5.1.4, the catalyst loading method has a large effect on the efficiency of the photocatalytic process. In terms of photoreactor design and geometry, some researchers attempted to implement monolithic and fiber optic designs to maximize the catalyst dispersion. These designs have not yet been slightly explored regarding HPs for photocatalytic CO_2_ reduction in specific. However, one example of such structures can be that of Li et al. The group reported successfully incorporating CsPbBr_3_ QDs into a silica/alumina monolith and developing a highly photostable system with a PLQY of almost 90%.^151^ The improvement in efficiency was explained by the increase in surface area and dispersion of the QDs on the monolith. Monolithic perovskite tandem solar cells have also been reported, indicating the prospects of applying such structures for photocatalytic CO_2_ reduction applications.^176^

**Figure 20:**
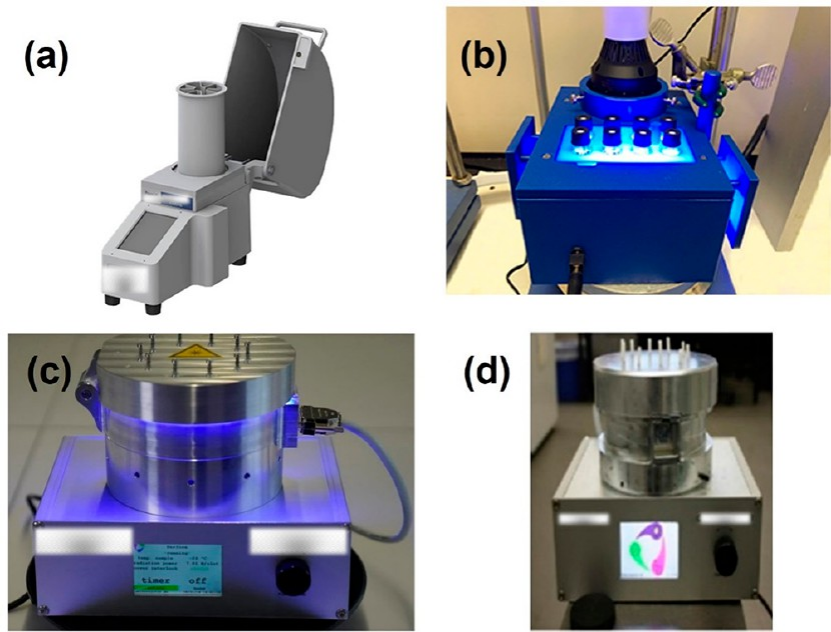
Different commercial photoreactors. (a) Photoreactor m2 (Penn Photon Devices, LLC), (b) EvoluChem PhotoRedOx Box (HepatoChem), (c) air-cooled TAK120 system (HK Testsysteme GmbH), and (d) liquid-cooled TAK120-LC system (HK Testsysteme GmbH).

Even with optimized reactor setups, the overall process can be time-consuming owing to the time needed to evacuate the air, purge CO_2_, and perform the reaction. For this reason, different commercial photoreactors can be used to perform quick screening tests to compare different photocatalysts or conditions simultaneously.^177^Figure 20 shows different existing commercial photoreactors with different features but similar functions. Using these devices, multiple reactions can be performed simultaneously by illuminating a group of vials. The designs differ in terms of the employed cooling system, light configuration, or vial capacity but all ensure a consistent and homogeneous distribution of light.

### 5.3 Control Experiments

As mentioned in Section 5.1, slight changes in any of the main reaction conditions or disturbances to the system might lead to significant variations in the obtained results. Combined with differences in reactor setup and photocatalyst synthetic procedures, it is often quite difficult to compare results reported in the literature and to know all the details of every setup. To eliminate any doubts associated with the experimental procedure and setup, control experiments are usually performed to ensure that the obtained products are strictly due to photocatalytic activity. The main control experiments often used to assess the catalyst performance involve conducting repeated tests in the absence of the electron donor, CO_2_, light, or the catalyst itself. These tests may help to detect any interferences from the setup or chemicals used during the photocatalyst synthetic procedure.

Perhaps the main control experiments are related to identifying the source of the carbon products. As can be observed from the examples displayed in Section 3, the most common synthetic procedures of HPs involve the use of organic solvents and capping ligands. Therefore, to ensure that any carbon products are solely due to the reduction of CO_2_, the reaction must be performed in an inert environment instead.^42^ For example, performing their photocatalytic reaction on MAPbI_3_@PCN-221(Fe*x*) NCs in a nitrogen atmosphere, Wu et al. were no longer able to observe the production of CO and CH_4_.^87^ When conducting their experiments in an Ar/H_2_O environment, Xu et al. noticed that their materials still produced large amounts of CO in the inert environment. Replacing the capping ligands used in the synthesis process with glycine led to the reduction of CO production. This led the group to believe that CO previously observed was from capping ligand degradation and not CO_2_. To confirm the stability of their catalyst when modified with glycine instead of capping ligands, the group performed ^13^CO_2_ tracer experiments that confirmed its production from the photocatalytic reduction of CO_2_ and not alternative sources further highlighting the stability of their catalyst.^178^

**Figure 21:**
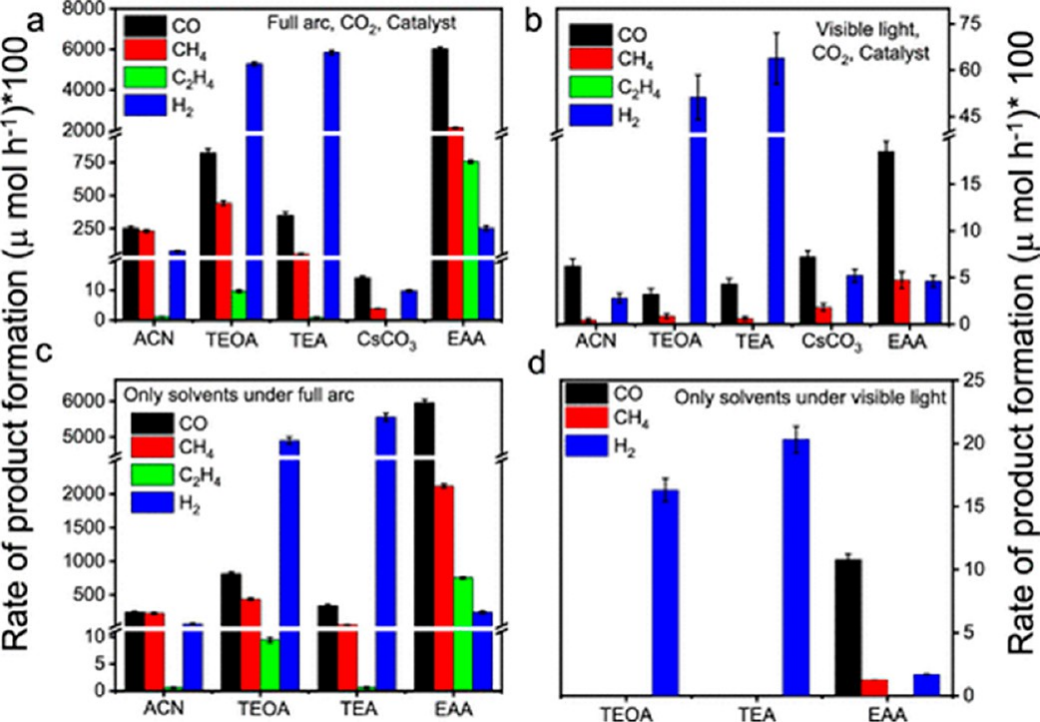
Rate of production of Pt-TiO_2_@g-C_3_N_4_ composites in different solvents under (a) UV–visible and (b) visible light. No catalyst control experiments performed under (c) UV–visible and (d) visible light. Reproduced from ref (164). Copyright 2021, American Chemical Society.

The significant impact of residual organic solvents can be better assessed when examining liquid-phase photocatalytic reactions. Das et al. highlighted this by conducting an assessment of solvent selection for photocatalytic CO_2_ reduction tests by a TiO_2_/g-C_3_N_4_ and BCN/CsPbBr_3_ nanocomposites performed in the liquid phase under, both, visible and UV light. As depicted in Figure 21, the group found that even in the absence of a catalyst (Figure 21a–d) significant production of all gases was still observed when using acetonitrile (ACN), triethanolamine (TEOA), triethylamine (TEA), and ethyl acetate (EEA) when illuminated.^164^

The group was able to prove that the photolysis of some organic solvents can produce H_2_, CO, CH_4_, and C_2_H_4_ in the absence of a catalyst. Their observations further strengthen the need for control tests when performing photocatalytic reactions to avoid reporting an overestimation of catalyst performance and misleading results.

### 5.4 Challenges in Characterization

To understand the crystal structure, morphology, and optoelectronic properties of HPs, various characterization techniques have been employed such as TEM, SEM, XRD, UV–vis, X-ray photoelectron spectroscopy (XPS), and many more. This section will highlight some of the main challenges observed when different techniques are used to characterize HPs.

**Figure 22:**
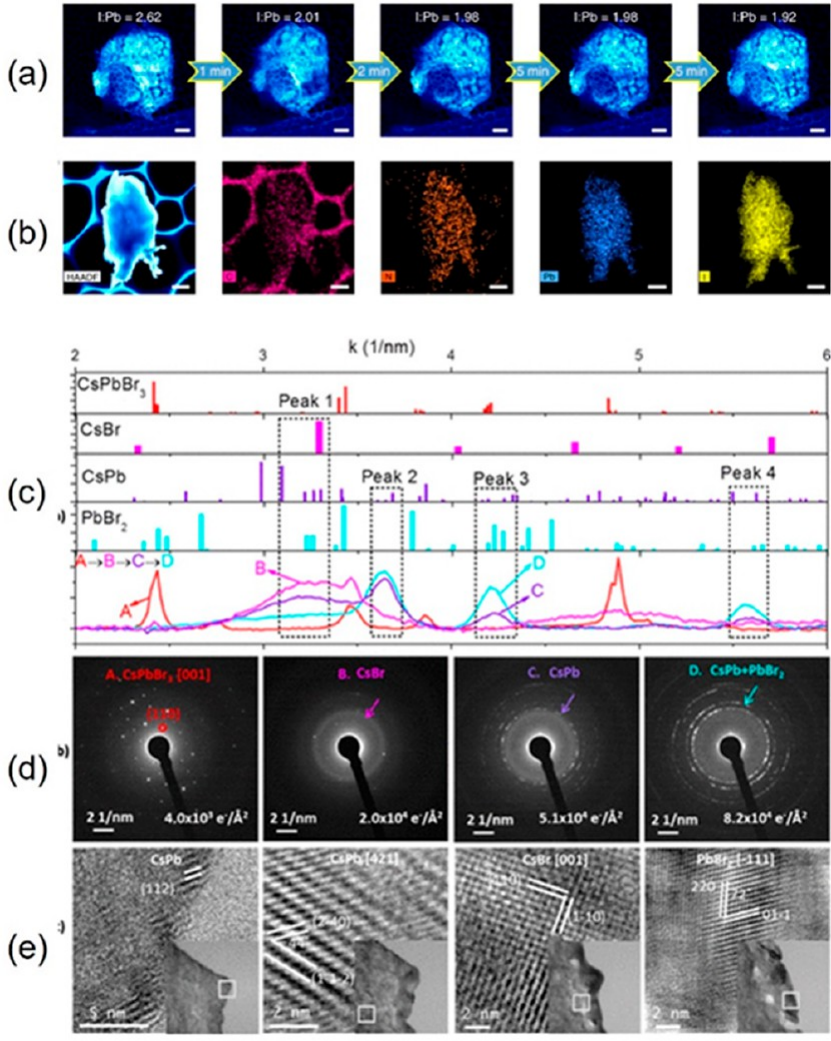
(a) SEM images of MAPbI_3_ with the atomic ratio for I/Pb from EDX mappings and (b) STEM image and EDX mappings of the sample for quantitative analysis. Reproduced with permission from ref (183). Copyright 2018, Springer Nature. (c, d) SAED patterns (A–D) of a single nanosheet at −120 °C and their azimuthal integration compared with the reference cards for the orthorhombic CsPbBr_3_ phase (ICSD: 97851), CsBr (ICSD: 236387), CsPb (ICSD: 627071), and PbBr_2_ (ICSD: 202134). (e) Zoomed-in view of the white-boxed regions in high-resolution TEM (HRTEM) images. Reproduced from ref (184). Copyright 2017, American Chemical Society.

Although electron microscopy characterization techniques such as SEM or HRTEM provide valuable information about the morphology, particle size, and structure, high intensity electron beams have proven to also cause damage and degradation of the materials.^179^ Most explored HP is the MAPbI_3_ perovskite which was proven to degrade back into PbI_2_ under the effect of electron illumination.^180,181^ Rothmann et al. observed that the {110} reflections corresponding to MAPbI_3_ obtained through TEM disappeared during the microscopy process when using a rapid acquisition rate.^182^ A similar observation was made by Chen et al. using SEM coupled with EDX mapping. The group was able to monitor the degradation of MAPbI_3_ to PbI_2_ through the decrease in the I/Pb ratio with time during data acquisition (Figure 22a,b).^183^Figure 22c–e show SAED patterns of CsPbBr_3_ nanosheets obtained sequentially at increasing irradiation doses. Dang et al. were able to demonstrate the decomposition of CsPbBr_3_ nanosheets and nucleation into CsBr, CsPb, and PbBr_2_ crystalline domains.^184^ Several research papers have tackled the challenges related to TEM analysis of HPs in more detail.^185−188^

Photoemission spectroscopy (PES) characterization techniques such as XPS and ultraviolet photoemission spectroscopy (UPS) are methods typically used to assess the surface chemical composition and electronic properties of HPs. Such techniques can help researchers indicate if the precursors involved in forming the HP have successfully interacted, test if surface modifications have indeed occurred, and understand the photocatalytic mechanism of the semiconductor.^189^ Like TEM analysis, PES techniques have also been a challenging characterization technique for HPs. Some of the main challenges include the formation of metallic Pb due to the reduction of Pb^2+^ in the perovskite structure caused by its instability upon the exposure to high energy X-ray beams.^190^

**Figure 23:**
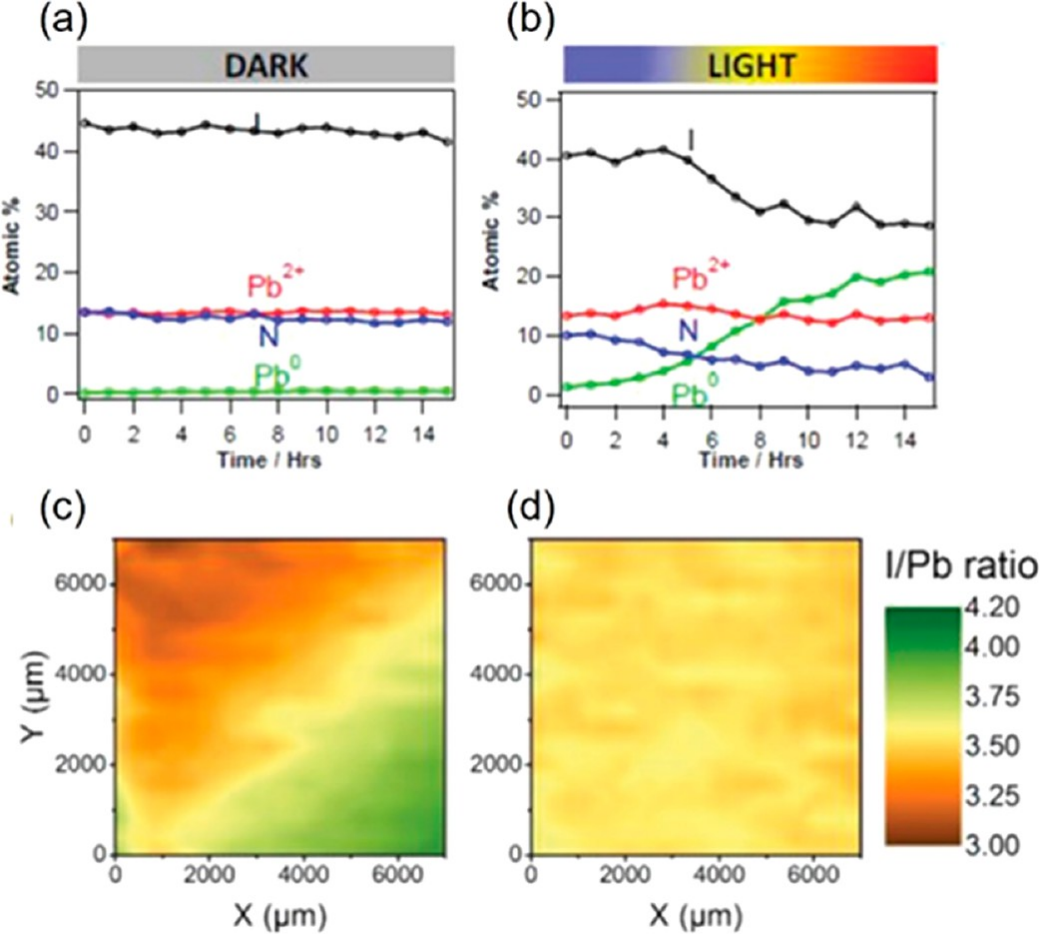
Variation of the elemental ratios on the surface of MAPI crystals under (a) dark and (b) light conditions with time. Reproduced with permission from ref (191). Copyright 2018, Royal Society of Chemistry Maps of I/Pb ratio of the perovskite films after storage (c) in N_2_ atmosphere and (d) under vacuum for 50 h. Reproduced from ref (192). Copyright 2018, American Chemical Society.

Another challenge faced when conducting PES measurements is caused by the need for UHV environments to conduct the tests. These conditions can often alter the surface composition of HPs due to the volatile nature of some of their components.^189^ Both Das et al. and Sun et al. performed studies to understand the effects of UHV conditions on the stability of MAPbI_3_ films. Figure 23a and b show the variation in the elemental concentration of MAPbI_3_ films with time under UHV in darkness and under illumination. The analysis shows that the material seems stable under UHV conditions unless illuminated.^191^ On the other hand, Figure 23c and d show how the I/Pb ratio decreases more when stored under UHV compared to the N_2_ atmosphere of a glovebox.^192^ In addition to that, the intrinsic instability of HPs and their sensitivity to moisture and O_2_ could lead to discrepancies in the results obtained from PES analysis.^189^

## 6 Summary and Outlook

In summary, we have provided an overview of halide perovskite (HP)-based photocatalysts, with more focus on their development for CO_2_ reduction reactions. Having prominent optical and electronic properties, HPs are promising candidates for CO_2_ photocatalytic reactions. Key factors influencing photocatalysis using HPs have been identified as intrinsic and extrinsic to the properties of HPs. For instance, the stability of HPs is an important intrinsic factor highly influencing photocatalytic activity and applications. A variety of methods which can be applied to maintain moisture, thermal and photostability of HPs during days have been listed such as formation of composites with more hydrophobic components; however, it remains challenging to achieve stabilities over longer times (e.g., weeks). Another intrinsic factor is the band structure of HPs. It is extremely tunable and methods allowing band gap engineering of HPs have been reported such as halide composition. Further research is required to engineer photocatalytic systems that provide enough photovoltage for CO_2_ redox reactions to occur with higher production rates (>1 mmol g^–1^ h^–1^) along with higher stability (>1 month). The morphology and structural properties of HPs significantly influence the band energies and hence photocatalytic properties. The HP properties also influence other factors such as charge separation, adsorption and desorption of reactants and products, and selectivity for more valuable products such as CO over CH_4_.

Extrinsic to the HPs, reaction conditions and reactor design strongly influence the photocatalytic results. The most important aspects that affect photocatalytic reactions were identified and highlighted as follows. The reaction temperature and pressure can heavily impact the adsorption–desorption equilibrium of the reactants. In addition to that, the wavelength of the incident light, its intensity, and duration can affect the solar fuel production since they are directly related to the number of photons taking part in the reaction. For HPs, the choice of adequate light properties is critical because it might as well have an impact on the stability of the photocatalyst, holder, and reactants. Loading the catalyst in the reactor is another important step, it is required to ensure that the surface area exposed to light is maximized. Reduction rates can be further enhanced using hole scavengers such as methanol, triethanolamine, or triethylamine but such research does not make progress on the use of most the convenient and abundant reductants such as water. Finally, another key factor is the sample synthesis and preparation before reaction, which can lead to excessive adventitious carbon on the surface of the photocatalyst affecting the results and interpretation.

The design of the photoreactor setup can limit the number of conditions one might need to carry out to fully understand the mechanism of the reactions being performed. For this reason, a complete and dynamic setup must allow for changes in temperature, pressure, light properties, and reaction phase (liquid or gas). Numerous setups have been constructed and optimized over the years, however, the general criteria for designing a successful gas-phase photoreactor are light transparency in the light spectrum range, purging options with the needed reactant mixture, and sampling mechanism for gas analysis. It is evident that the interplay of the different reaction conditions and the overall setup introduces numerous variables that affect our understanding of the process. With that in mind, it is crucial for the research field that future work puts more emphasis on standardized methodologies for photocatalytic measurements and in reporting detailed process and reaction conditions, including control tests that confirm photocatalytic activity.

We envisage that the application of HPs in the photocatalytic field will continue to grow, exploiting their unique optoelectronic properties. There are many opportunities in the development of lead-free halide perovskites and their derivatives, as well as in the formation of heterojunctions with novel materials that boost charge separation and CO_2_ adsorption and reactivity. Advances in HP-based solar cells and in characterization techniques such as in-operando ones will feed in crucial information to design more stable and photocatalytically active HP materials. Finally, novel reactor designs, for example those ones that exploit photocatalytic sheets or concentrated sunlight, together with the help of high-throughput approaches and machine learning, will accelerate the progress toward more efficient devices that achieve commercialization in a short future.
